# Adult neurogenesis in the short-lived teleost *Nothobranchius furzeri*: localization of neurogenic niches, molecular characterization and effects of aging

**DOI:** 10.1111/j.1474-9726.2011.00781.x

**Published:** 2012-04

**Authors:** Eva Terzibasi Tozzini, Mario Baumgart, Giorgia Battistoni, Alessandro Cellerino

**Affiliations:** 1 Fritz Lipmann Institute for Age Research, Leibniz InstituteJena, Germany; 2 Scuola Normale SuperiorePisa, Italy

**Keywords:** neuronal stem cells, aging, doublecortin, microRNA, gliosis, animal model

## Abstract

We studied adult neurogenesis in the short-lived annual fish *Nothobranchius furzeri* and quantified the effects of aging on the mitotic activity of the neuronal progenitors and the expression of glial fibrillary acid protein (GFAP) in the radial glia. The distribution of neurogenic niches is substantially similar to that of zebrafish and adult stem cells generate neurons, which persist in the adult brain. As opposed to zebrafish, however, the *N. furzeri* genome contains a doublecortin (DCX) gene. Doublecortin is transiently expressed by newly generated neurons in the telencephalon and optic tectum (OT). We also analyzed the expression of the microRNA miR-9 and miR-124 and found that they have complementary expression domains: miR-9 is expressed in the neurogenic niches of the telencephalon and the radial glia of the OT, while miR-124 is expressed in differentiated neurons. The main finding of this paper is the demonstration of an age-dependent decay in adult neurogenesis. Using unbiased stereological estimates of cell numbers, we detected an almost fivefold decrease in the number of mitotically active cells in the OT between young and old age. This reduced mitotic activity is paralleled by a reduction in DCX labeling. Finally, we detected a dramatic up-regulation of GFAP in the radial glia of the aged brain. This up-regulation is not paralleled by a similar up-regulation of S100B and Musashi-1, two other markers of the radial glia. In summary, the brain of *N. furzeri* replicates two typical hallmarks of mammalian aging: gliosis and reduced adult neurogenesis.

## Introduction

The continued production of neurons throughout adulthood, in at least in some circumscribed brain regions, has been observed in all vertebrate species so far studied. In mammals, adult neurogenesis is restricted to two principal stem cell niches in the telencephalon: the subependymal zone (or subventricular zone, SVZ) which generates GABAergic and dopaminergic interneurons of the olfactory bulb and the subgranular zone (SGZ) of the dentate gyrus. These areas contain adult neuronal stem cells (aNSCs) of glial phenotype, which are able to self-renew. aNSCs generate an intermediate precursor, which gives rise to a transient amplifying neuroblast and finally to neurons (Gage *et al.*, 2008). These different stages can be identified by a combination of morphological criteria and the expression of molecular markers. For example, aNSCs in the hippocampus are characterized by the expression of SOX2, glial fibrillary acid protein (GFAP), and activated Notch signaling (Lugert *et al.*, 2010), whereas the amplifying neuroblast expresses a combination of PSA-NCAM, doublecortin (DCX), and the mitotic marker Ki-67 (Gage *et al.*, 2008). Doublecortin expression (but not expression of mitotic markers) persists for some time in young terminally differentiated neurons (Brown *et al.*, 2003). Neurons generated during adulthood are functional, integrate into existing circuits, and are proposed to be involved into some specific aspects of behavioral plasticity (van Praag *et al.*, 2002; Gage *et al.*, 2008).

Adult neurogenesis is a highly plastic process, which can be enhanced by environmental stimulation and physical exercise (Kempermann *et al.*, 1997; van Praag *et al.*, 1999) and it is modulated by epigenetic mechanisms, microRNAs (Cheng *et al.*, 2009; Yoo *et al.*, 2009; Ma *et al.*, 2010), and growth factors.

Neurogenesis in the SGZ decreases exponentially with age in rodents (Kuhn *et al.*, 1996; Ben Abdallah *et al.*, 2010), dogs (Pekcec *et al.*, 2008), and humans (Knoth *et al.*, 2010). This decay is initiated during early postnatal life, and, in adults, neuronal production in the SGZ is < 10% of what observed during puberty (Ben Abdallah *et al.*, 2010; Knoth *et al.*, 2010). Age-dependent decrease in neurogenesis is observed in SVZ as well, but it is less dramatic than in the SGZ (Luo *et al.*, 2006).

Decreased adult neurogenesis is the consequence of quiescence of aNSCs, which are still present, but not active, in the old brain and can be re-activated by physiological and pathological stimuli (Lugert *et al.*, 2010).

In contrast to mammals, adult neurogenesis is extensive in teleost fish and is observed basically in all brain regions (Zupanc & Horschke, 1995; Ekstrom *et al.*, 2001). Systematic anatomical studies in zebrafish and medaka (Adolf *et al.*, 2006; Grandel *et al.*, 2006; Kuroyanagi *et al.*, 2010) have identified at least 16 different neurogenic niches. In fish, aNSCs are mostly associated with the ventricular system. In the fish telencephalon, aNSCs have the typical morphology of radial glia and share several molecular markers of mammalian aNSCs (Ganz *et al.*, 2010; Marz *et al.*, 2010). A pallial and subpallial neurogenic niche can be identified in fish and these are homolog to the SGZ and SVZ of mammals (Adolf *et al.*, 2006; Mueller & Wullimann, 2009). Analysis of viral tracing has directly demonstrated that adult radial glial is self-renewing and pluripotent, proving its stem cell identity (Rothenaigner *et al.*, 2011). aNSCs in the optic tectum (OT) (Ito *et al.*, 2010) and cerebellum (Kaslin *et al.*, 2009) are not of glial nature and express neuroepithelial markers.

Studies on zebrafish used young adults of age 3–6 months and maximum reported lifespan for this species is 66 months (Gerhard *et al.*, 2002) and there are no quantitative reports on age-dependent modulation of adult neurogenesis. As fish show substantial growth during adult life, it is unclear to what extent adult neurogenesis is responsible for neuronal turnover or rather reflects addition of new cells in the context of continuous brain growth.

Gliosis, measured as up-regulation of GFAP in astroglia, is a well-described hallmark of mammalian aging (Riddle, 2007; Park *et al.*, 2009). In fish, however, radial glia is the predominant glia type (Cuoghi & Mola, 2009) and retains its neurogenic function in adulthood. It is therefore of interest to explore whether also radial glia undergoes gliosis as function of age.

To investigate the effects of aging on adult neurogenesis in teleosts, we used a novel animal model: the short-lived fish *Nothobranchius furzeri*. This species has a captive lifespan of 3–7 months depending on the strain (Terzibasi *et al.*, 2008) and it is an emerging model organism for biological investigations into aging (Hartmann *et al.*, 2009; Terzibasi *et al.*, 2009; Graf *et al.*, 2010; Di Cicco *et al.*, 2011; Hartmann *et al.*, 2011). In particular, we demonstrated that *N. furzeri* undergoes age-dependent neurodegeneration and cognitive impairments (Valenzano *et al.*, 2006a, b; Terzibasi *et al.*, 2009), which in mammals often correlate with reduced adult neurogenesis (Gage *et al.*, 2008).

Aims of the present paper were the following:

1. To localize neurogenic niches in the adult brain of *N. furzeri.*
2. To investigate the expression of conserved molecular markers of neurogenesis in this species.
3. To quantify the effects of age on adult neurogenesis in the OT.
4. To study the effects of aging on the radial glia.

## Results

### Distribution of neurogenic niches in the brain of *Nothobranchius furzeri*

#### Whole mounts

**Fig. 1:**
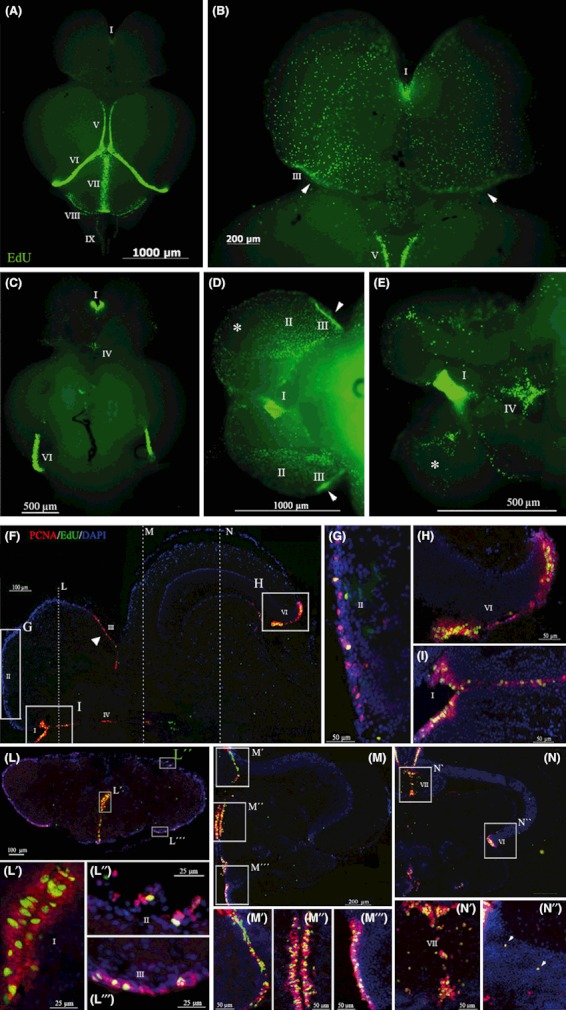
Illustration of neurogenic niches. (A–E) Whole mount (WM) overview of Edu+ cells in 7-week-old *Nothobranchius furzeri* visualized 4 h after intraperitoneal injection. Dorsal view of the entire brain (A) and telencephalic region (B). (C–E) Ventral view of the entire brain, low magnification (C), and two different orientations at higher magnification (D, E). Neurogenic niches are identified by Latin numerals: I = telencephalic proliferative niche corresponding to the subpallial region visible in panel A – II, III = telencephalic proliferative niches corresponding to the pallial regions visible in panel B, D, E – IV = preoptic proliferative niche visible in panel E were the optic nerves were removed – V = rostro-dorsal part of the proliferative niche in the optic tectum (OT), visible in panel A, B (partially) – VI = caudal part of the proliferative niche in the OT, visible in panel A, C – VII = medial proliferative niche of the cerebellum, visible in panel A – VIII = caudal proliferative niche of the cerebellum, visible in panel A – IX = caudal proliferative niche along the roof of the IV ventricle, visible in panel A. *The region of the telencephalic surface devoid of proliferating cells, possibly of pial origin. Arrowheads in panels B and D indicate areas of higher concentration of proliferating cells in the caudal margin of niche III. F-N: horizontal and coronal brain sections illustrating PCNA+ (red) and Edu+ (green) cells in 6-week-old *N. furzeri* visualized 4 h after intraperitoneal injection. The approximate level corresponding to coronal sections represented in panels L, M, N is shown as dotted lines on a dorsal view of the brain in the inset of panel F. (F) Emi-section of brain in horizontal view: all telencephalic proliferative niches described in (A–E) are visible (I, II, III), as well as the preoptic niche (IV) and the niche of the caudal margin of the OT (VI). (G–I) Magnification of areas corresponding to the boxed regions in panel F. (L) Coronal section of the brain at median telencephalic level: the three proliferative niches are visible (I, II, III) and magnified in panels L^I^, L^II^, L^III^. (M) Coronal section of the brain at rostral level of the OT: the dorso-medial tectal niche bordering with the torus longitudinalis (tl) is visible (V) and three different areas of the proliferative niches distributed along the ventricular wall are magnified in panels M^I^, M^II^, M^III^. (N) Coronal section of the brain at median level of the OT: the medial tectal niche bordering with the cerebellum (crb) is visible (VII), as well as the niche of the tectal ventro-caudal margin (VI); regions VII is magnified in panels N′; in N″, a partial magnification of region VI shows few double-labeled cells scattered within the subventricular gray zone of the OT (arrowheads). DAPI nuclear counterstaining is visualized in blue.

To label mitotically active cells, we injected the nucleotide analog 5-ethynil-2′deoxyuridin (EdU). Whole-mount detection of Edu+ cells showed a widespread distribution in the brain of young (5–7 weeks old) subjects 4 h after injection (Fig. 1A–E). We concentrated on those areas located in telencephalon, OT, and cerebellum, which were subject of in-depth studies in zebrafish and medaka (Adolf *et al.*, 2006; Kaslin *et al.*, 2009; Alunni *et al.*, 2010; Ito *et al.*, 2010).

In the telencephalon, three distinct regions can be recognized: A niche of tightly packed high proliferating cells, which starts quite sharply at the rostro-ventral conjunction of the two telencephalic hemispheres (Fig. 1B–E, region I). This region has a subpallial origin, is homologous to the SVZ of mammals (Mueller & Wullimann, 2009), and is observed in the telencephalon of other teleost species (Zupanc & Horschke, 1995; Ekstrom *et al.*, 2001; Adolf *et al.*, 2006; Grandel *et al.*, 2006; Alunni *et al.*, 2010; Kuroyanagi *et al.*, 2010). Neurogenic cells are also scattered on the dorsal and lateral surfaces of the telencephalon in various teleost species. Because of developmental eversion of the telencephalic vesicles, this region of pallial origin is facing the third ventricle and it is proposed to be homologous to the subgranular neurogenic niche of mammals (Mueller & Wullimann, 2009). In *N. furzeri*, the pallial neurogenic region can be subdivided into two different areas region II and III (Fig. 1B,D): Region II is placed dorsally and rostrally and shows a much lower level of proliferation as compared to region I; region III is placed ventro-caudally and shows a level of proliferation intermediate between regions I and II. Region II and region III are separated by a ventro-lateral patch of telencephalic surface devoid of proliferative cells (Fig. 1D, indicated with *). This area is observed also in zebrafish and is suggested to correspond to the pial rather than the ventricular surface (Mueller & Wullimann, 2009). Proliferation in region III is concentrated on its ventro-caudal portion (Fig. 1B,D arrowhead). An area of high proliferative activity is observed in the preoptic segment of the ventricular system (region IV, Fig. 1C,E). Intense neurogenic activity was observed in the dorsomedial, posterior, and ventral margins of the OT (regions V and VI; Fig. 1A,C). A clearly visible neurogenic niche is located along the midline surface of the cerebellum (Fig. 1A, region VII). Further distinct niches visible in whole-mount preparations are located at the caudal margin of the cerebellum (Fig. 1A, region VIII) and lining the margin of the fourth ventricle, (Fig. 1B, region IX).

Sagittal overviews of the *N. furzeri* brain labeled with EdU are shown in Fig. S1 (Supporting Information).

#### Telencephalon

Double-labeling with EdU and the proliferation marker proliferating cell nuclear antigen (PCNA) was performed in coronal and horizontal sections of 5- to 7-week-old subjects 4 h after injection (Fig. 1F–N, Figs S2 and S3). Neurogenic niches identified by the presence of EdU+ cells always correspond to regions labeled with PCNA. In telencephalon, regions I, II, and III can be readily appreciated in horizontal and coronal sections. Region I (Fig. 1I) extends from the niche visible in whole-mount preparations along the third ventricle in caudal direction. Analysis of sections confirms the presence of a region II (Fig. 1G) and a region III. The concentration of dividing cells in the caudal margin of the telencephalon in region III is clearly visible in horizontal sections (Fig. 1F, white arrowhead). In zebrafish, the pallial neurogenic niche contains slow-cycling precursors and the subpallial niche contains faster-dividing precursors (Adolf *et al.*, 2006; Ganz *et al.*, 2010). These can be distinguished based on the percentage of colocalization between PCNA and EdU. High colocalization is diagnostic of fast cycle (Adolf *et al.*, 2006). Conventional microscopy is suggestive of a much higher co-localization in the subpallial area I (Fig. 1F–I, L′–L‴). Confocal microscopy confirms a higher proportion of double-labeled cells in the subpallial region I (Fig. S3).

#### Ventricular system

Caudal to region IV in the preoptic region, neurogenic niches were observed widespread along the ventricle, in the hypothalamus and caudally to the medulla oblongata and spinal cord (e.g. Fig. 1M). We do not attempt here to subdivide this into more defined areas.

#### Optic tectum

**Fig. 5:**
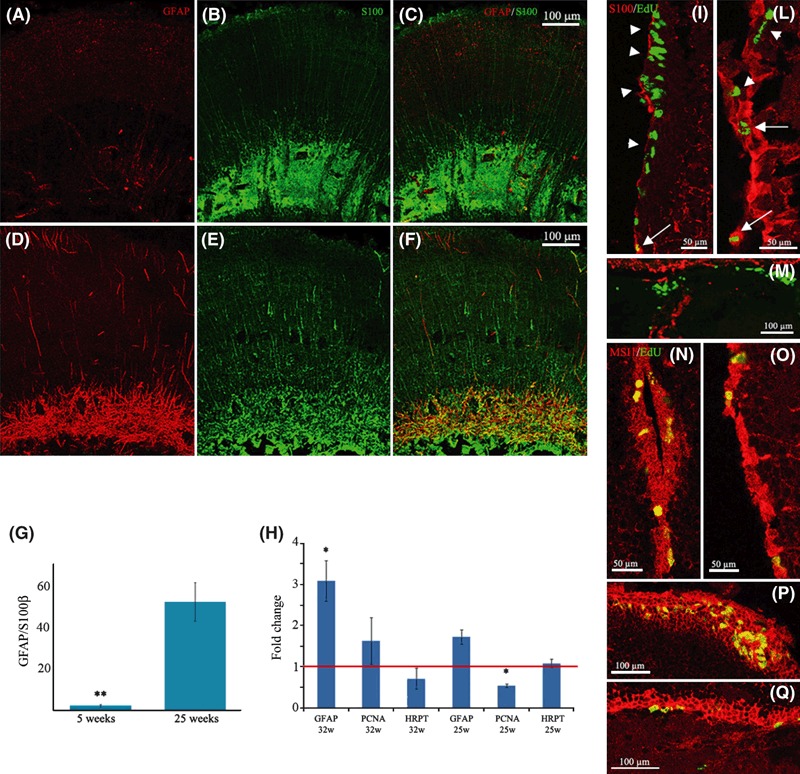
Comparison of double-staining for S-100 (green) and glial fibrillary acid protein (GFAP) (red) in the central area of the optic tectum (OT) of young (5-week-old) vs. old (25-week-old) *Nothobranchius furzeri* brain; confocal images (projection of seven optical sections for 6 μm thickness). (A, B, C) Representation of single GFAP (A), S-100B (B), and merged staining GFAP+S-100B (C) in the tectal central region of a 5-week-old fish. (D, E, F) Representation of single GFAP (D), S-100B (E), and merged staining GFAP+S-100B (F) in the tectal central region of a 25-week-old fish; S-100B shows intense staining in both young and old subjects, whereas GFAP staining is weak in the young tissue but undergoes a strong age-dependent up-regulation. Scale bar reported in panel A is representative for all the other panels (B–F). (G) Quantification of labeling intensity of GFAP. Intensity is expressed as pixels over threshold and is normalized on the number of pixels positive for S100B. Error bars are standard errors of means. **P < 0.01, Student’s *t*-test. Four animals for each age were used. (H) Quantification of mRNAs per real time. Fold changes are relative to 5-week-old controls. Values are normalized with respect to TBP. Error bars are standard errors of means. *P < 0.05, permutation-based test, REST software. Number of animal: 5-week-old N = 8, 27-week-old N = 8, 32-week-old N = 6. (I–M) Double-labeling for 5-ethynil-2′deoxyuridin (EdU) (green) and S100B (red). Single confocal planes, double-labeled cells are indicated by arrowheads and cells labeled by EdU only by arrows. (I) Subpallial neurogenic niche, note lack of co-localization. (L) Pallial neurogenic niche. (M) Germinal layer of the OT. (N–Q) Double-labeling for EdU (green) and Msi-1 (red). Single confocal planes. Note complete co-localization in all areas. (N) subpallial, (O) pallial, (P) germinal layer of the OT, (Q) radial glia of the OT. See Fig. S6 (Supporting Information) for single channels.

Region V corresponds to the medial margin of the OT and is placed at the border with the *torus longitudinalis*. Proliferating cells are present on both sides of this border in the OT and the *torus longitudinalis*. Region V shows a typical U-shape in coronal sections (Fig. 1M). Region VI corresponds to the posterior and ventral margins of the OT (Fig. 1H,N). Scattered Edu+, PCNA-positive cells could be detected in the subventricular gray zone of the OT (Figs 1N″ and 5Q).

#### Cerebellum

Region VII represents the cerebellar neurogenic niche. This niche was characterized in detail in zebrafish (Kaslin *et al.*, 2009). In *N. furzeri*, region VII has the shape of a dorsal and a ventral wedge of highly active cells connected by a rod of dividing cells in the *valvula cerebelli* (Fig. 1N,N′). The size of the dorsal wedge increases and that of the ventral wedge decreases in caudal direction in *corpus cerebelli* and in the most posterior portion only the dorsal wedge is visible.

A complete rostro-caudal series of coronal sections to better appreciate the full extent of these neurogenic niches is presented in Fig. S2 (Supporting Information).

### Newly generated cells are long-living and differentiate into neurons

#### Long-term survival

**Fig. 2:**
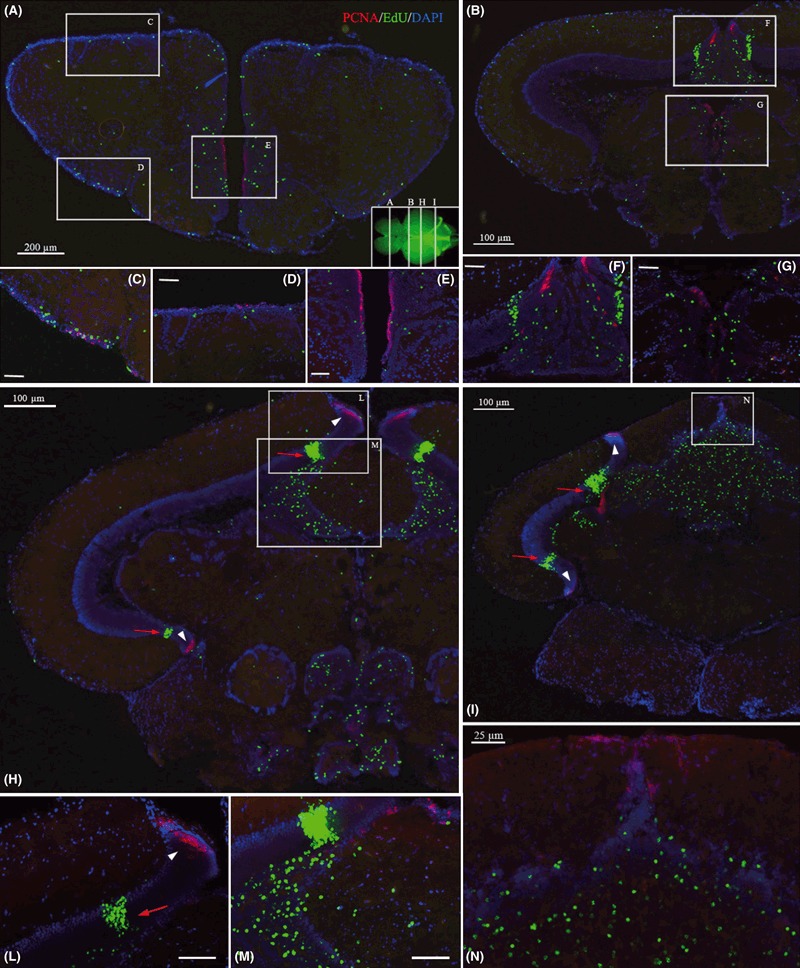
Coronal sections of 11-week-old *Nothobranchius furzeri* stained for PCNA (red) and 5-ethynil-2′deoxyuridin (EdU) (green), 5 weeks after intraperitoneal EdU injection. Approximate position of the sections in panels A, B, H, I along the rostro-caudal axis of the brain is shown on top view of a whole-mount brain in the inset of panel A. (A) Coronal section of the telencephalon in median position: after 5 weeks, EdU+ cells migrate centrifugally from the ventricular and dorso-lateral margins into the parenchyma, outside of the PCNA+ proliferative regions. (B) Coronal section of a rostral portion of the optic tectum (OT): in the antero-dorsal margin (magnification in panel F) EdU+ cells form a strip into the tectal parenchyma well distinct from the PCNA+ cells area. (C–E) Magnifications of the dorsal, lateral, and ventricular margin of the telencephalic section, respectively, represented in pictures A. (F, G) Magnifications of the antero-dorsal tectal margin and the III ventricle, respectively: in both cases, EdU+ cells migrate after 5 weeks into the parenchyma. (H, I) Coronal sections of the central and caudal portions of the OT: the antero-dorsal and postero-ventral strips of EdU+ cells into the parenchyma can be appreciated as well (red arrows); centripetally migrating EdU+ cells can be observed also in the *torus longitudinalis* (picture B, magnified panel F). A very large number of EdU+ cells can be found in the granule cell layer of the *valvula cerebelli* (picture H, magnified panel M) and *corpus cerebelli* (picture I, magnified panel N). Scale bar for magnification panels C, D, E, F, G, L, and M are the same as indicated in panel N.

To prove the persistence of cells generated during adult life, we analyzed animals 5 weeks after a single injection of EdU. Median lifespan for the MZM-04/10 strain is 27 weeks in our fish colony (Terzibasi *et al.*, 2009), so this interval covers a significant proportion of the adult life for this species. In all brain regions, EdU-labeled cells persisted and were found into the brain parenchyma at some distance from the neurogenic niches of origin visualized with PCNA (Fig. 2). This is best appreciated in the OT posterior margin, where the newly generated cells from a dense strip (Fig. 2F,H–M). The distance between this EdU+ strip (red arrows in Fig. 2H,I) and the PCNA+ germinal zone (white arrowhead in Fig. 2H,I) represents the marginal growth of the OT within this period. In the cerebellum, neurogenesis is extensive and cells generated during adulthood migrate in the granule cell layer (Fig. 2H–I,M–N). At visual inspection, it seems that more cells are generated and integrated into the cerebellum than in any other region of the *N. furzeri* brain, in line with the extensive cerebellar neurogenesis reported in other fish species (Zupanc & Horschke, 1995; Ekstrom *et al.*, 2001; Adolf *et al.*, 2006; Kaslin *et al.*, 2009; Kuroyanagi *et al.*, 2010).

#### Neuronal differentiation

To study the fate of the newly generated cells, we sacrificed animals 1 week after a single injection of EdU and performed a double-labeling with the neuronal marker HuC/D (Marusich *et al.*, 1994). Sections were then analyzed by confocal microscopy. As expected, extensive co-localization was observed in telencephalon, OT, and cerebellum (Fig. S4).

### Molecular characterization of the neuronal precursors

#### MicroRNA expression

MicroRNAs are emerging as important regulators of neurogenesis and in particular miR-9 and miR-124 (Leucht *et al.*, 2008; Cheng *et al.*, 2009; Yoo *et al.*, 2009). miR-124 is expressed by differentiated neurons while miR-9 was reported, in zebrafish, to be up-regulated in the regions where adult neurogenesis occurs (Kapsimali *et al.*, 2007).

**Fig. 3:**
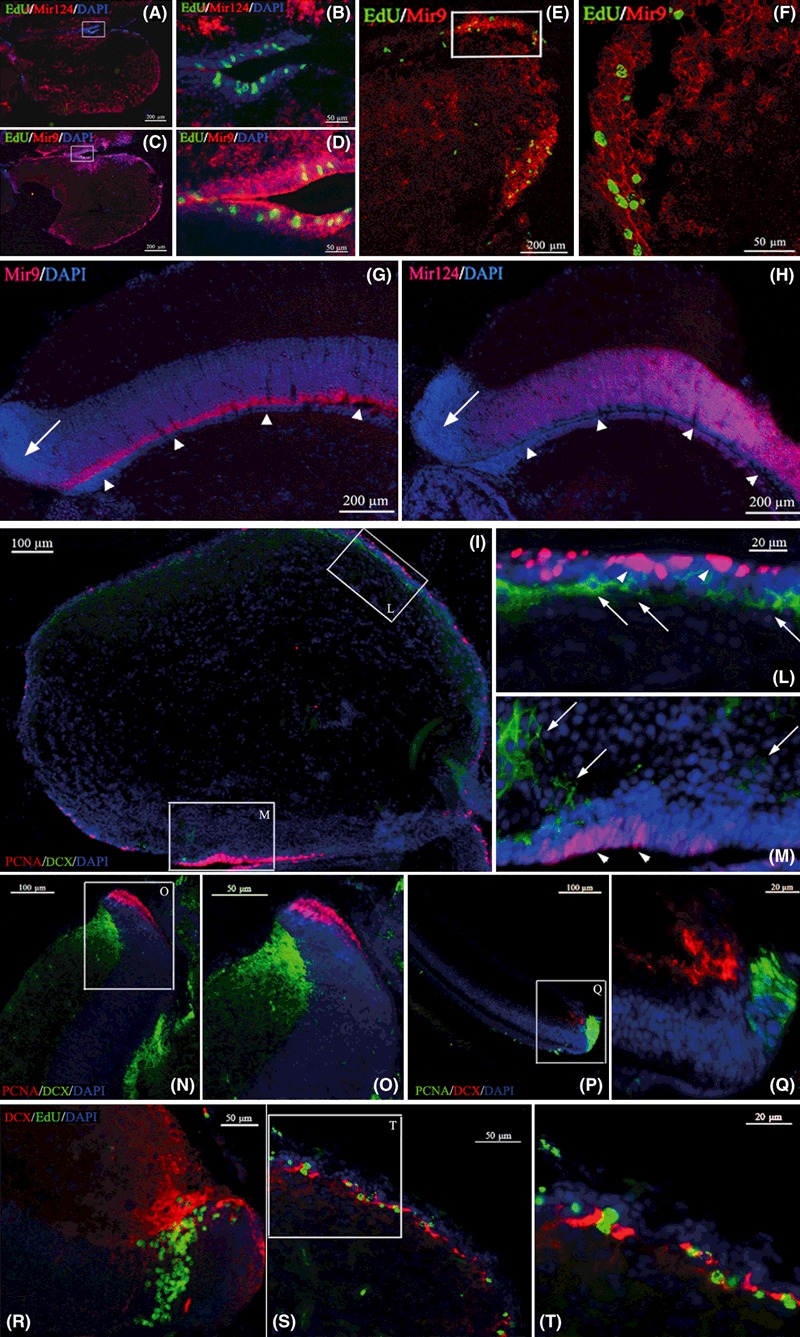
*In situ* hybridization for miR-124 and miR-9 (A–H) in the telencephalic and tectal structures of 7-week-old *Nothobranchiu*s *furzeri* (young adult), and double-staining for doublecortin (DCX)/PCNA andDCX/5-ethynil-2′deoxyuridin (EdU) (I-T). (A) Overview of the right telencephalon in horizontal section where EdU+ cells (green) and miR-124+ cells (red) are visualized. (B) Magnification of the pallial neurogenic niche boxed in A is shown in panel B; the exclusion of miR-124 labeling from the EdU+ cells area is apparent. (C) Overview of the right telencephalon in horizontal section where EdU+ cells (green) and miR9+ cells (red) are visualized. (D) Magnification of the telencephalic subpallial neurogenic area boxed in C. (E) Confocal image of a single optical plane, horizontal section of the pallial neurogenic area. Magnification of the region boxed in E is shown in panel F. (G, H) *In situ* hybridization labeling in the optic tectum for miR-9 (G) and miR-124 (H). Note the exclusion of miR-124 and miR-9 as well from the germinal layer (arrow) and the complementary labeling pattern of miR-124 and miR-9 in the radial glia (arrowheads). (N–T) Horizontal sections of 7-week-old *N. furzeri* (young adult) double-labeled for DCX and PCNA (I–Q) or DCX and EdU, 1-2 weeks after intraperitoneal injection (R–T). (I) Horizontal overview of the right telencephalon stained for PCNA (red), DCX (green). (M, N) Magnification of the telencephalic pallial and subpallial neurogenic regions, respectively. Note that in the pallial neurogenic region (L), a strip of DCX+ cells is present immediately adjacent to the PCNA+ cells marginal stratum. In contrast, in the subpallial niche (M) only DCX+ fibers can be detected. (N, O) Representation at low (N) and high (M) magnification of the ventro-posterio tectal margin: a strip of DCX+ neuronal processes (green) is present a few cell diameter medial to the PCNA+ cells (red) in the germinal layer. (P, Q) Representation at low (P) and high (Q) magnification of the retinal ciliary marginal zone: a concentration of DCX+ neuronal processes (red) is present medial from the PCNA+ cells (green) proliferative margin. (R) Representation of the ventro-posterio tectal margin stained for DCX (red) and EdU (green, 1 week postinjection): the strip of EdU+ cells is aligned with the strip of DCX+ processes. (S, T) Representation at low (S) and high (T) magnification of the latero-posterior telencephalic margin stained for DCX (red) and EdU (green, 2 weeks postinjection): the majority of EdU+ cells after 2 weeks correspond with the strip of DCX+ cells. The nuclear DAPI is visualized as blue staining in panel B, D, G, H. (T) Scale bars are directly indicated in the panels: bar in the same for pictures L and M. DCX, doublecortin.

MicroRNAs are extremely conserved in their sequence. A preliminary study of small RNA sequencing (M. Baumgart, M. Grothe, M. Platzer, A. Cellerino, paper in preparation) revealed that the sequences of mature miR-124 and miR-9 in *N. furzeri* are identical to the sequences of *Danio rerio*. We therefore used locked nucleic acid (LNA) *in situ* probes designed for *D. rerio*. miR-124 expression was detected in nearly all neurons throughout the adult brain, but not in the neurogenic niches. This was particularly evident in the subpallial area I of the telencephalon (Fig. 3A,B). The expression pattern of miR-9 in the telencephalon was complementary to that of miR-124 and was concentrated in the neurogenic niches (Fig. 3C,D). Confocal microscopy (Fig. 3E,F) revealed partial colocalization of miR-9 and EdU 4 h postinjection, indicating that miR-9 is expressed in mitotically active neuronal progenitors.

In the OT, expression of miR-124 (Fig. 3G) was lower in the neurogenic niche (arrow) and in the neighboring area that contains newly differentiated cells. Expression of miR-124 was specifically reduced in a strip of cells in the most medial portion of the OT (arrowheads). This area corresponds to the location of radial glia (Fig. 5, see below), where expression of miR-9 is particularly high (Fig. 3H). miR-9, however, was not up-regulated in the germinal layer of the OT (arrow), indicating that miR-9 is not associated with neuroepithelial progenitors. Single-channel visualizations of *in situ* hybridization for miR-124 and miR-9 in the telencephalic structure (Fig. 3B,D,F) are shown in Fig. S6 (Supporting Information, A/B, D/E, G/H, respectively).

#### Doublecortin

In mammals, neuroblasts and newly generated neurons express DCX (Brown *et al.*, 2003). Because of excellent labeling of neuronal processes, this marker is widely used to quantify neurogenesis (Pekcec *et al.*, 2008; Ben Abdallah *et al.*, 2010; Knoth *et al.*, 2010). A DCX ortholog is present neither in the assembled zebrafish genome nor in the collections of zebrafish expressed sequence tags. We recovered a transcript, which we call NfuDCX, in a database of *N. furzeri* transcripts (A. Petzold, K. Reichwald, N. Hartmann, A. Cellerino, C. Englert, M. Platzer, manuscript in preparation). NfuDCX shows 86.5% aminoacidic sequence similarity to mouse DCX (Data S2). To confirm that NfuDCX is the ortholog of DCX, we performed a phylogenetic analysis aligning all DCX and DCX-like sequences present in the ENSEMBL database. NfuDCX clusters with all other DCX sequences from other vertebrate species and not with DCX-like kinase 1, or DCX-like kinase 2 (Fig. S5). This analysis also revealed that DCX is present in the genomes of the Actynoptegyian fishes *Gastrosteleus aculeatus* (stickleback), *Tetraodon nigroviridis*, and *Takifugu rubipes* (pufferfish) (Table S1, Fig. S5).

Immunolabeling with DCX in the telencephalon (Fig. 3I–M, green) revealed a strip of strongly labeled cells 3–4 cell diameters in distance from the telencephalic surface that is adjacent, but does not overlap, with the ribbon of PCNA+ progenitors (Fig. 3I–M,red). Double-labeling with PCNA and DCX in the OT (Fig. 3N–O, PCNA red and DCX green) and the retina (Fig. 3P–Q, PCNA green and DCX red) revealed that a column of processes labeled for DCX can be found juxtaposed to the germinal margins of active neurogenesis. One week after EdU injections, the column of DCX-labeled processes in the OT (Fig. 3R, red) was in register with the front of EdU+ cells close to the germinal margin (Fig. 3R, green). Two weeks postinjection, the column of DCX-labeled process is found more medial with respect to the strip of EdU+ cells. In the telencephalon, the timing of DCX expression was slower. Only few cells were double-labeled with DCX (red) and EdU (green) 1 week after injection (data not shown), but double-labeling of EdU and DCX is observed 2 weeks after injection (Fig. 3S-T).

**Fig. 4:**
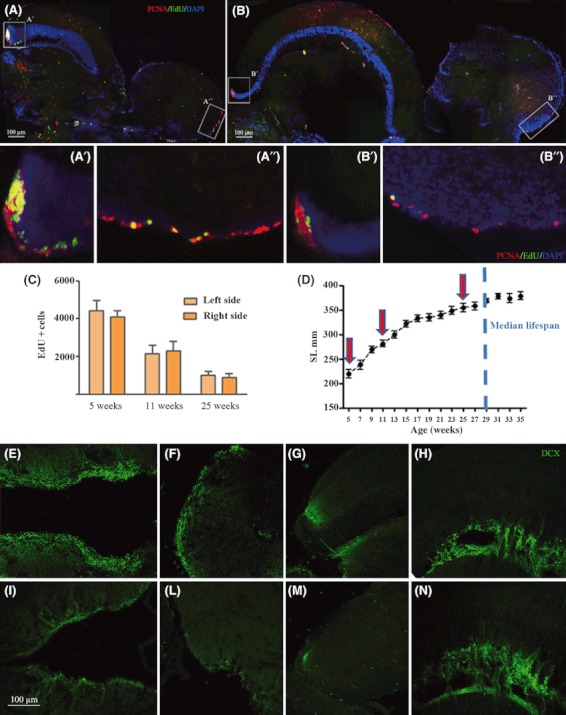
Horizontal sections double-labeled for PCNA+ (red) and Edu+ (green) in the ventro-posterior margin (niche VI) of the optic tectum (OT): 5- vs. 25-week-old *Nothobranchius furzeri*, visualized 4 h after intraperitoneal 5-ethynil-2′deoxyuridin (EdU) injection. (A, B) Hemisection of young (5-week-old, (A) vs. aged (25-week-old, (B) brain in horizontal view; regions undergoing evident age-dependent reduction in PCNA+ and EdU+ labeling are better appreciated in the correspondent magnification in the lower panel strip. (A′) the OT posterior margin niche of a young animal (5 weeks old) is represented, showing an intense double-labeled proliferative cells in the germinal layer. By comparison, in the old subject represented in B′), few double-labeled PCNA+/EdU+ cells can be detected in the PCNA+ germinal layer. (C) Histogram reporting unbiased estimation of EdU+ cells into the tectal germinal layer at three different age-steps. Estimates for left and right *tecta* are reported separately. Five animals per time point were analyzed, and error bars represent standard deviations. (D) The panel on the right represents the growth curve of *N. furzeri* males. The analyzed age-steps for the quantification are marked by red arrows. Forty-five animals were measured to produce this curve. Error bars represent standard errors of means. Scale bar for magnification panels (A′, A″, B″) is the same, as indicated in panel B″. (E–N) Comparison of doublecortin (DCX) expression (green) in telencephalic and tectal areas of young (5-week-old) vs. old (25-week-old) *N. furzeri* brain; confocal images (projection of seven optical sections for 6 μm thickness) A. (D) Representation of the telencephalic subpallial (E) and pallial (F) neurogenic niches, and the germinal layer (G) and central region (H) of the OT of a 5-week-old brain: DCX+ processes are present in all telencephalic and tectal margins, as well as in the central tectum. Representation of the subpallial (I) and pallial (L) neurogenic niches of the telencephalon, and the germinal layer (M) and central region (N) of the OT of a 25-week-old brain: DCX+ processes are clearly reduced in the neurogenic areas (I–M) when compared to the corresponding regions (E–G) in the 5-week-old brain. On the other hand, DCX staining in the central tectum shows similar pattern and intensity at both ages and can be regarded as a control for labeling quality.

It should be remarked that DCX does not represent a marker of newly generated neurons throughout the brain. Doublecortin expression was not observed in association with the subpallial neurogenic niche (area I) (Fig. 3L), and strong DCX labeling was observed in the olfactory nerve, in fibers entering the olfactory bulb (data not shown) and in fibers entering the medial portion of the OT (Fig. 4H,N).

### Effects of aging

#### Neurogenesis

To quantify the effects of age on neurogenesis, 25-week-old animals were analyzed 4 h after EdU injection and compared to 11-week- and 5-week-old animals. Neurogenic niches are still present in old fish, but the mitotic activity in telencephalon and OT is visibly reduced (Fig. 4A,B). We quantified the number of Edu+ cells in the germinal layer of the OT using unbiased stereological estimates (see Experimental procedures). A significant age-dependent reduction in proliferating cells was detected (Fig. 4C, anova, *P* < 0.0001). The absolute number of EdU+ cells monotonically decreased between 5, 11, and 25 weeks. Reduced neurogenesis resulted also in visibly reduced DCX staining in the telencephalon (Fig. 4I–L) and on the posterior margin of the OT (Fig. 4M) of 25-week-old fish when compared to 5-week-old fish (Fig. 4E–G). No differences were seen in the central region of the OT (Fig. 4H–N), where DCX expression is not associated with neurogenesis.

Comparison of neurogenesis decay with somatic growth reveals that there is no direct relationship between these two processes (Fig. 4D). Indeed, neurogenesis is already reduced at age 11 weeks, when fish are still in the growing phase, and it is present – even if reduced – at 25 weeks of age, when the fish have practically ceased to grow.

#### Gliosis, Musashi-1, S100B, and GFAP

Age-dependent up-regulation of GFAP is a well-characterized hallmark of aging in mammals (Riddle, 2007; Park *et al.*, 2009). We therefore studied age-dependent regulation of glial markers in *N. furzeri*.

The expression of glial markers in telencephalic aNSCs was studied in great detail in zebrafish (Ganz *et al.*, 2010; Marz *et al.*, 2010). In zebrafish, pallial aNSCs, but not the aNSCs in the subpallial region, expresses the typical glial markers S100B and GFAP. Cells on the germinal zone of the OT do not express glial markers and have a neuroepithelial phenotype (Ito *et al.*, 2010).

Glial fibrillary acid protein is not detected in the telencephalon of young *N. furzeri*. Only faint labeling of GFAP+ radial fibers is observed in the OT of young *N. furzeri* (Fig. 5A). S100B is expressed in the neurogenic telencephalic areas II and III in cells with the typical morphology of radial glia, but not in the subpallial area I (Fig. 5I–L, Fig. S8). In the OT, S100B marks the radial glia, which constitutes the most medial margin of the OT and is excluded from the germinal layer (Fig. 5M, Fig. S8). Double-labeling with EdU 4 h postinjection and confocal analysis revealed a partial colocalization, demonstrating that at least some S100B+ cells in the pallium are mitotically active (Fig. 5L white arrows, Fig. S8).

Mushasi-1 (Msi-1) is an RNA-binding protein necessary for neurogenesis and enriched in aNSC (Sakakibara *et al.*, 1996). The expression domain of S100B largely overlaps with that of Msi-1. Msi-1, however, also labels the germinal layer of the subpallial neurogenic niche and OT (Fig. 5N–Q), which are not labeled for S100B. At a cellular level, confocal analysis revealed complete co-localization between Msi-1 and Edu 4 h postinjection, revealing that Msi-1 labels both the radial glia and the amplifying neuroblasts (Fig. 5N–Q, Fig. S8). This analysis also revealed that scattered EdU+ cells in the OT are Msi-1+ (Fig. 5Q).

A dramatic up-regulation of GFAP immunoreactivity is observed in the OT of 25-week-old fish (Fig. 5D) when compared to 5-week-old fish (Fig. 5A). A dense network of fibers running tangentially to the medial margin of the OT is observed, and also, radial processes become strongly positive. These changes are not accompanied by up-regulation of S100B (Fig. 5B,E) or Msi-1 (Fig. S5), although GFAP immunoreactivtiy co-localizes with S100B (Fig. 5C,F) and Msi-1 (Fig. S7). Quantification of GFAP/S100B ratio revealed a ∼ 20-fold increase between 5-weeks and 25-weeks of age (Fig. 5G). Up-regulation of GFAP mRNA in the entire brain could also be detected by real-time PCR (Fig. 5H).

## Discussion

In the present paper, we analyzed the niches of adult neurogenesis in *N. furzeri*. We found that the neurogenic niches have a similar distribution to that described in detail in zebrafish. In particular, in the telencephalon of both species, there is a clear distinction between a less active pallial and a more active subpallial niche (Adolf *et al.*, 2006; Ganz *et al.*, 2010; Marz *et al.*, 2010). The pallial zone contains slow-cycling mitotically active radial glia positive for S100B, while the subpallial zone contains fast-cycling aNSCs lacking S100B labeling.

Organization of stem cell niches in the OT of *N. furzeri* is also conserved and almost identical to what described in medaka (Alunni *et al.*, 2010; Ito *et al.*, 2010): a clearly defined, continuous, and very active germinal layer is observed in the lateral, posterior, and inferior border of the OT. Progenitors in the OT are not labeled with S100B antibodies. In addition, a sparse neurogenic activity can be observed throughout the medial margin of the OT. This position corresponds to the location of the radial glia in the OT and double-labeling of EdU, and Msi-1 indicates that also the radial glia in the OT is mitotically active, although at a much lower level as compared to the telencephalon. This is a very similar situation to what described in the retina, where there is a neuroepithelial ciliary marginal zone which can produce all cells with exception of rods and neurogenic Müller glia which produces only rods. It remains to be investigated whether radial glia in the OT produces a specific neuronal type or not.

We further characterized the neurogenic niches in telencephalon and OT to define expression of conserved molecular markers. The microRNAs, miR-9 and miR-124, are known to control neurogenesis and neuronal differentiation (Leucht *et al.*, 2008; Cheng *et al.*, 2009; Yoo *et al.*, 2009; Bonev *et al.*, 2011). Here, we report that miR-9 is highly expressed in the radial glia in telencephalon and OT but not in the neuroepithelial precursors of the OT. miR-124, on the other hand, is specifically excluded in neuronal precursors and newly generated neurons. This result is in line with the expression domains reported in adult zebrafish brain (Kapsimali *et al.*, 2007) and suggests that miR-9 controls the activity of the neurogenic radial glia, while miR-124 is necessary for differentiation of postmitotic neurons.

The RNA-binding protein Musashi-1 (Msi-1) was found to be expressed in all neurogenic niches in telencephalon and OT and its expression was not restricted to radial glia. All cells labeled with EdU after short intervals were found also to be positive for Msi-1; therefore, this essential neurogenic gene is expressed both in the aNSCs and in the amplifying progenitors in *N. furzeri*.

An interesting finding was the identification of DCX in *N. furzeri*. This gene is necessary for adult neurogenesis in mammals (Jin *et al.*, 2010) and is the marker of choice to label newly generated neurons (Brown *et al.*, 2003), but it is absent from the zebrafish genome. In *N. furzeri*, DCX labels newly generated neurons in pallium and OT and this strongly suggests a conservation of DCX function between mammals and Actinopterygian fishes. Doublecortin expression in *N. furzeri* is dramatically down-regulated with age, as in all mammalian species studied thus far (Gage *et al.*, 2008; Pekcec *et al.*, 2008; Ben Abdallah *et al.*, 2010; Knoth *et al.*, 2010).

The most important finding of the present paper is the demonstration of a strong age-dependent regulation of neurogenesis in *N. furzeri*. This age-dependent regulation does not directly correlate with somatic growth and was observed already during early adult life, as observed in mammals (Pekcec *et al.*, 2008; Ben Abdallah *et al.*, 2010; Knoth *et al.*, 2010).

Gliosis is a widespread phenomenon in the aging mammalian brain, which is easily detected as up-regulation of GFAP (Riddle, 2007; Park *et al.*, 2009). Aging-dependent gliosis was not investigated in any fish species so far. We could detect massive gliosis in the radial glia of the OT as a consequence of aging in *N. furzeri*. Gliosis is the typical response of the brain to injury and is observed in many neurodegenerative diseases. Age-dependent gliosis is therefore a further indication of age-dependent neurodegeneration in *N. furzeri*. This increase in GFAP is not paralleled by a similar dramatic increase in S100B or Msi-1, indicating that the number of radial glia does not change dramatically but these cells up-regulate GFAP expression.

In summary, we have shown conserved aspects of adult neurogenesis in *N. furzeri* both in the location of neurogenic niches and in the expression of molecular markers. More importantly, we report that *N. furzeri* shows age-dependent decrease in neurogenesis and age-dependent gliosis. Intervention studies in aging mammals are associated with high costs and long experimental times. Our data qualify *N. furzeri* as an alternative model organism to study mechanisms underlying age-dependent regulation of neurogenesis and gliosis.

## Experimental procedures

Detailed experimental procedures are provided as Data S1.

### Animal experimentation

All experiments were performed on group-house *N. furzeri* of the MZM-04/10 strain. Details can be found in Terzibasi *et al.* (2009). The protocols of fish maintenance were approved by the local authority in the State of Thuringia (Veterinär- und Lebensmittelüberwachungsamt).

### Immunohistochemistry and *in situ* hybridization

Immunohistochemistry was performed using commercially available antibodies (Table S2). In situ hybridization was performed using LNA probes labeled with DIG at 5′ and 3′ at an hybridization temperature of 42°C. EdU (50 μL of a 10 μm solution) was injected intraperitoneally and detected according to manufacturer’s instruction (Invitrogen, Grand Island, NY, USA).

### Cloning of GFAP and quantitative PCR

Glial fibrillary acid protein (Data S3) was cloned using Rapid Amplification of cDNA Ends (RACE; Clonthech, Mountain View, CA, USA), and expression levels were quantified using the Qiagen protocols on a Rotorgene (Qiagen, Hilden, Germany).

### Unbiased estimates of cell numbers

To quantify EdU+ cells, brains were serially sectioned and every third section was analyzed. To count cells, two confocal planes at a distance of 4 μm were superimposed and cells present in both planes were excluded. Unbiased cell estimates were so derived:

Let the ordered series of sections for a specimen be 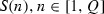, and let the sampling density be 1/*X*, where *Q* = *M* × *X*, then a random number 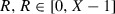 is extracted and every section 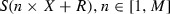 is analyzed. Then, the unbiased estimate of cell numbers 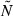 can be calculated by the formula


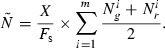



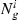
 = number of profiles present only in the reference (upper) plane of section *i*, 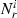 = number of profiles present only in the bottom plane of section *i*, *F*_S_ = fraction of total section volume contained between reference and bottom planes.

## Supporting Information

**Fig. S1** Sagittal views of EdU-labeled brains.

**Fig. S2** Complete coronal series of EdU PCNA labeling.

**Fig. S3** Confocal images PCNA+ EdU.

**Fig. S4** Double-labeling EdU+ HuC/D.

**Fig. S5** Phylogenetic tree of DCX and DCX-like kinases.

**Fig. S6** *In situ* hybridization for miR-124 and miR-9 (separated and merged channels).

**Fig. S7** Double-labeling Msi-1+ GFAP.

**Fig. S8** Double-labeling S100B+ EdU, Msi-1+ EdU, confocal images.

**Table S1** Accession numbers of DCX sequences.

**Table S2** List of antibodies.

**Data S1** Experimental procedures.

**Data S2** Alignment of NfuDCX with mouse DCX.

**Data S3** Alignment of NfuGFAP with mouse GFAP.

As a service to our authors and readers, this journal provides supporting information supplied by the authors. Such materials are peer-reviewed and may be re-organized for online delivery, but are not copy-edited or typeset. Technical support issues arising from supporting information (other than missing files) should be addressed to the authors.

## References

[b1] Adolf B, Chapouton P, Lam CS, Topp S, Tannhauser B, Strahle U, Gotz M, Bally-Cuif L (2006). Conserved and acquired features of adult neurogenesis in the zebrafish telencephalon. Dev. Biol.

[b2] Alunni A, Hermel JM, Heuze A, Bourrat F, Jamen F, Joly JS (2010). Evidence for neural stem cells in the medaka optic tectum proliferation zones. Dev. Neurobiol.

[b3] Ben Abdallah NM, Slomianka L, Vyssotski AL, Lipp HP (2010). Early age-related changes in adult hippocampal neurogenesis in C57 mice. Neurobiol. Aging.

[b4] Bonev B, Pisco A, Papalopulu N (2011). MicroRNA-9 reveals regional diversity of neural progenitors along the anterior-posterior axis. Dev. Cell.

[b5] Brown JP, Couillard-Despres S, Cooper-Kuhn CM, Winkler J, Aigner L, Kuhn HG (2003). Transient expression of doublecortin during adult neurogenesis. J. Comp. Neurol.

[b6] Cheng LC, Pastrana E, Tavazoie M, Doetsch F (2009). miR-124 regulates adult neurogenesis in the subventricular zone stem cell niche. Nat. Neurosci.

[b7] Cuoghi B, Mola L (2009). Macroglial cells of the teleost central nervous system: a survey of the main types. Cell Tissue Res.

[b8] Di Cicco E, Tozzini ET, Rossi G, Cellerino A (2011). The short-lived annual fish *Nothobranchius furzeri* shows a typical teleost aging process reinforced by high incidence of age-dependent neoplasias. Exp. Gerontol.

[b9] Ekstrom P, Johnsson CM, Ohlin LM (2001). Ventricular proliferation zones in the brain of an adult teleost fish and their relation to neuromeres and migration (secondary matrix) zones. J. Comp. Neurol.

[b10] Gage FH, Kempermann G, Song H (2008). Adult neurogenesis. Cold Spring Harbor Monograph Series.

[b11] Ganz J, Kaslin J, Hochmann S, Freudenreich D, Brand M (2010). Heterogeneity and Fgf dependence of adult neural progenitors in the zebrafish telencephalon. Glia.

[b12] Gerhard GS, Kauffman EJ, Wang X, Stewart R, Moore JL, Kasales CJ, Demidenko E, Cheng KC (2002). Life spans and senescent phenotypes in two strains of Zebrafish (*Danio rerio*. Exp. Gerontol.

[b13] Graf M, Cellerino A, Englert C (2010). Gender separation increases somatic growth in females but does not affect lifespan in *Nothobranchius furzeri*. PLoS ONE.

[b14] Grandel H, Kaslin J, Ganz J, Wenzel I, Brand M (2006). Neural stem cells and neurogenesis in the adult zebrafish brain: origin, proliferation dynamics, migration and cell fate. Dev. Biol.

[b15] Hartmann N, Reichwald K, Lechel A, Graf M, Kirschner J, Dorn A, Terzibasi E, Wellner J, Platzer M, Rudolph KL, Cellerino A, Englert C (2009). Telomeres shorten while Tert expression increases during ageing of the short-lived fish *Nothobranchius furzeri*. Mech. Ageing Dev.

[b16] Hartmann N, Reichwald K, Wittig I, Drose S, Schmeisser S, Luck C, Hahn C, Graf M, Gausmann U, Terzibasi E, Cellerino A, Ristow M, Brandt U, Platzer M, Englert C (2011). Mitochondrial DNA copy number and function decrease with age in the short-lived fish *Nothobranchius furzeri*. Aging Cell.

[b17] Ito Y, Tanaka H, Okamoto H, Ohshima T (2010). Characterization of neural stem cells and their progeny in the adult zebrafish optic tectum. Dev. Biol.

[b18] Jin K, Wang X, Xie L, Mao XO, Greenberg DA (2010). Transgenic ablation of doublecortin-expressing cells suppresses adult neurogenesis and worsens stroke outcome in mice. Proc. Natl. Acad. Sci. U S A.

[b19] Kapsimali M, Kloosterman WP, de Bruijn E, Rosa F, Plasterk RH, Wilson SW (2007). MicroRNAs show a wide diversity of expression profiles in the developing and mature central nervous system. Genome Biol.

[b20] Kaslin J, Ganz J, Geffarth M, Grandel H, Hans S, Brand M (2009). Stem cells in the adult zebrafish cerebellum: initiation and maintenance of a novel stem cell niche. J. Neurosci.

[b21] Kempermann G, Kuhn HG, Gage FH (1997). More hippocampal neurons in adult mice living in an enriched environment. Nature.

[b22] Knoth R, Singec I, Ditter M, Pantazis G, Capetian P, Meyer RP, Horvat V, Volk B, Kempermann G (2010). Murine features of neurogenesis in the human hippocampus across the lifespan from 0 to 100 years. PLoS ONE.

[b23] Kuhn HG, Dickinson-Anson H, Gage FH (1996). Neurogenesis in the dentate gyrus of the adult rat: age-related decrease of neuronal progenitor proliferation. J. Neurosci.

[b24] Kuroyanagi Y, Okuyama T, Suehiro Y, Imada H, Shimada A, Naruse K, Takeda H, Kubo T, Takeuchi H (2010). Proliferation zones in adult medaka (*Oryzias latipes*) brain. Brain Res.

[b25] Leucht C, Stigloher C, Wizenmann A, Klafke R, Folchert A, Bally-Cuif L (2008). MicroRNA-9 directs late organizer activity of the midbrain-hindbrain boundary. Nat. Neurosci.

[b26] Lugert S, Basak O, Knuckles P, Haussler U, Fabel K, Gotz M, Haas CA, Kempermann G, Taylor V, Giachino C (2010). Quiescent and active hippocampal neural stem cells with distinct morphologies respond selectively to physiological and pathological stimuli and aging. Cell Stem Cell.

[b27] Luo J, Daniels SB, Lennington JB, Notti RQ, Conover JC (2006). The aging neurogenic subventricular zone. Aging Cell.

[b28] Ma DK, Marchetto MC, Guo JU, Ming GL, Gage FH, Song H (2010). Epigenetic choreographers of neurogenesis in the adult mammalian brain. Nat. Neurosci.

[b29] Marusich MF, Furneaux HM, Henion PD, Weston JA (1994). Hu neuronal proteins are expressed in proliferating neurogenic cells. J. Neurobiol.

[b30] Marz M, Chapouton P, Diotel N, Vaillant C, Hesl B, Takamiya M, Lam CS, Kah O, Bally-Cuif L, Strahle U (2010). Heterogeneity in progenitor cell subtypes in the ventricular zone of the zebrafish adult telencephalon. Glia.

[b31] Mueller T, Wullimann MF (2009). An evolutionary interpretation of teleostean forebrain anatomy. Brain Behav. Evol.

[b32] Park SK, Kim K, Page GP, Allison DB, Weindruch R, Prolla TA (2009). Gene expression profiling of aging in multiple mouse strains: identification of aging biomarkers and impact of dietary antioxidants. Aging Cell.

[b33] Pekcec A, Baumgartner W, Bankstahl JP, Stein VM, Potschka H (2008). Effect of aging on neurogenesis in the canine brain. Aging Cell.

[b34] van Praag H, Kempermann G, Gage FH (1999). Running increases cell proliferation and neurogenesis in the adult mouse dentate gyrus. Nat. Neurosci.

[b35] van Praag H, Schinder AF, Christie BR, Toni N, Palmer TD, Gage FH (2002). Functional neurogenesis in the adult hippocampus. Nature.

[b36] Riddle DR (2007). Brain Aging: Models, Methods, and Mechanisms.

[b37] Rothenaigner I, Krecsmarik M, Hayes JA, Bahn B, Lepier A, Fortin G, Gotz M, Jagasia R, Bally-Cuif L (2011). Clonal analysis by distinct viral vectors identifies bona fide neural stem cells in the adult zebrafish telencephalon and characterizes their division properties and fate. Development.

[b38] Sakakibara S, Imai T, Hamaguchi K, Okabe M, Aruga J, Nakajima K, Yasutomi D, Nagata T, Kurihara Y, Uesugi S, Miyata T, Ogawa M, Mikoshiba K, Okano H (1996). Mouse-Musashi-1, a neural RNA-binding protein highly enriched in the mammalian CNS stem cell. Dev. Biol.

[b40] Terzibasi E, Valenzano DR, Benedetti M, Roncaglia P, Cattaneo A, Domenici L, Cellerino A (2008). Large differences in aging phenotype between strains of the annual fish *Nothobranchius furzeri*. PLoS ONE.

[b41] Terzibasi E, Lafrancois C, Domenici P, Hartmann N, Graf M, Cellerino A (2009). Effects of dietary restriction on mortality and age-related phenotypes in the short-lived fish *Nothobranchius furzeri*. Aging Cell.

[b42] Valenzano DR, Terzibasi E, Cattaneo A, Domenici L, Cellerino A (2006a). Temperature affects longevity and age-related locomotor and cognitive decay in the short-lived fish *Nothobranchius furzeri*. Aging Cell.

[b43] Valenzano DR, Terzibasi E, Genade T, Cattaneo A, Domenici L, Cellerino A (2006b). Resveratrol prolongs lifespan and retards the onset of age-related markers in a short-lived vertebrate. Curr. Biol.

[b44] Yoo AS, Staahl BT, Chen L, Crabtree GR (2009). MicroRNA-mediated switching of chromatin-remodelling complexes in neural development. Nature.

[b45] Zupanc GK, Horschke I (1995). Proliferation zones in the brain of adult gymnotiform fish: a quantitative mapping study. J. Comp. Neurol.

