# Supplementary figures and images for: Adult neurogenesis in the short-lived teleost Nothobranchius furzeri: localization of neurogenic niches, molecular characterization and effects of aging
Source: Aging Cell. 2012 Apr;11(2):241–51. doi: 10.1111/j.1474-9726.2011.00781.x (PMC3437507; doi:10.1111/j.1474-9726.2011.00781.x)

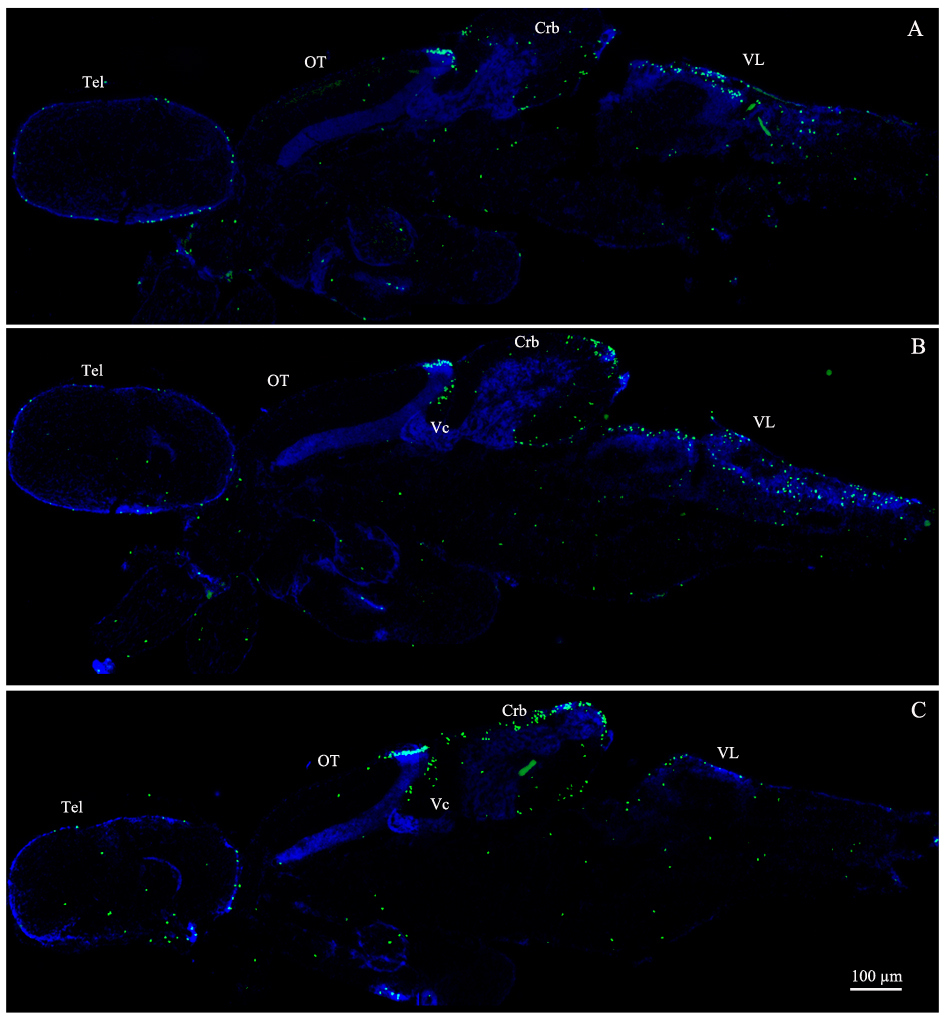

Supplement: Supplementary file 1 [file acel0011-0241-SD1.jpg]

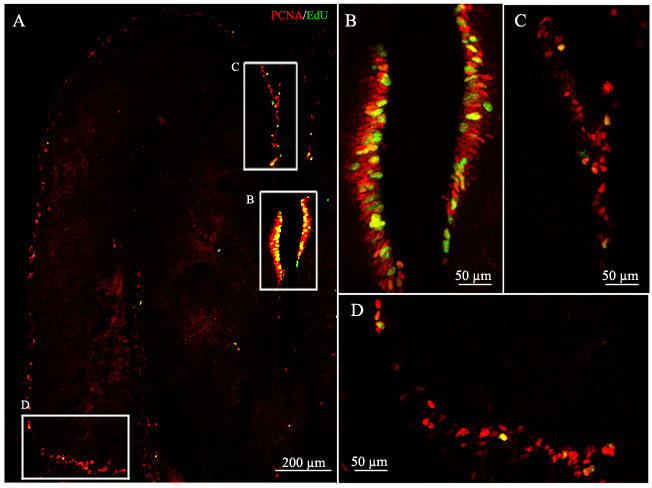

Supplement: Supplementary file 3 [file acel0011-0241-SD3.jpg]

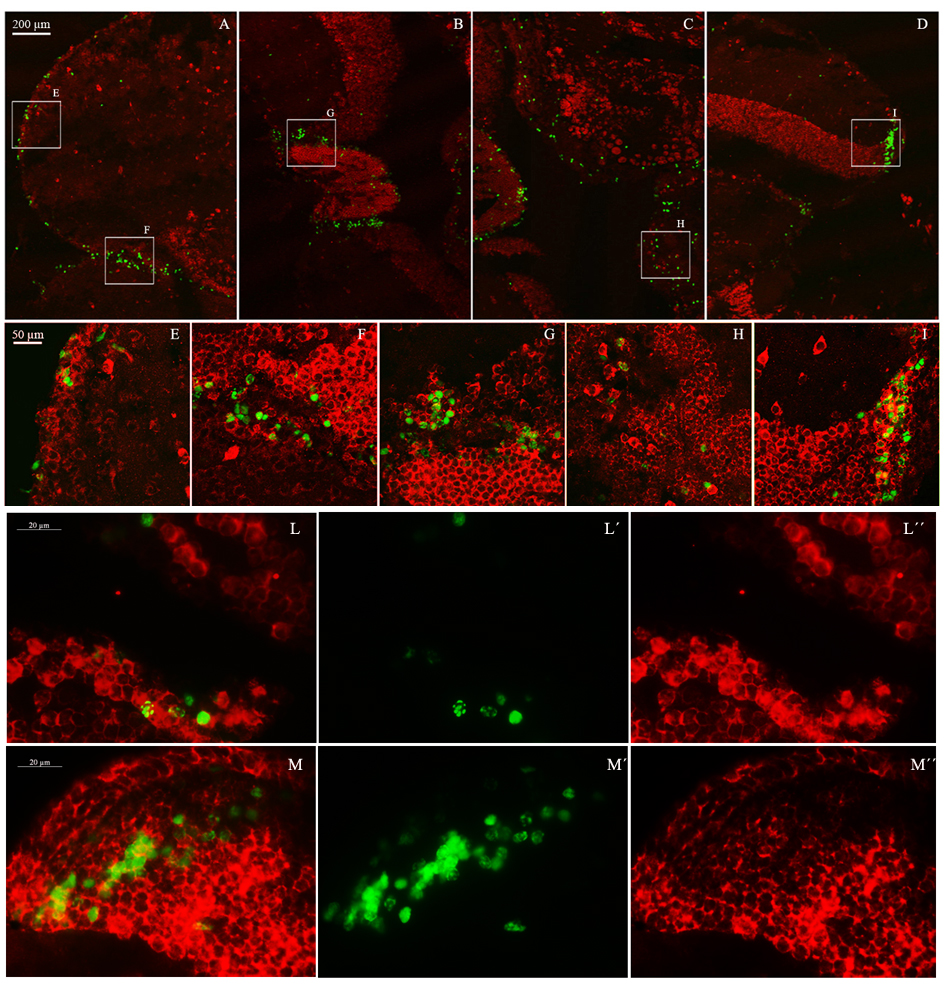

Supplement: Supplementary file 4 [file acel0011-0241-SD4.jpg]

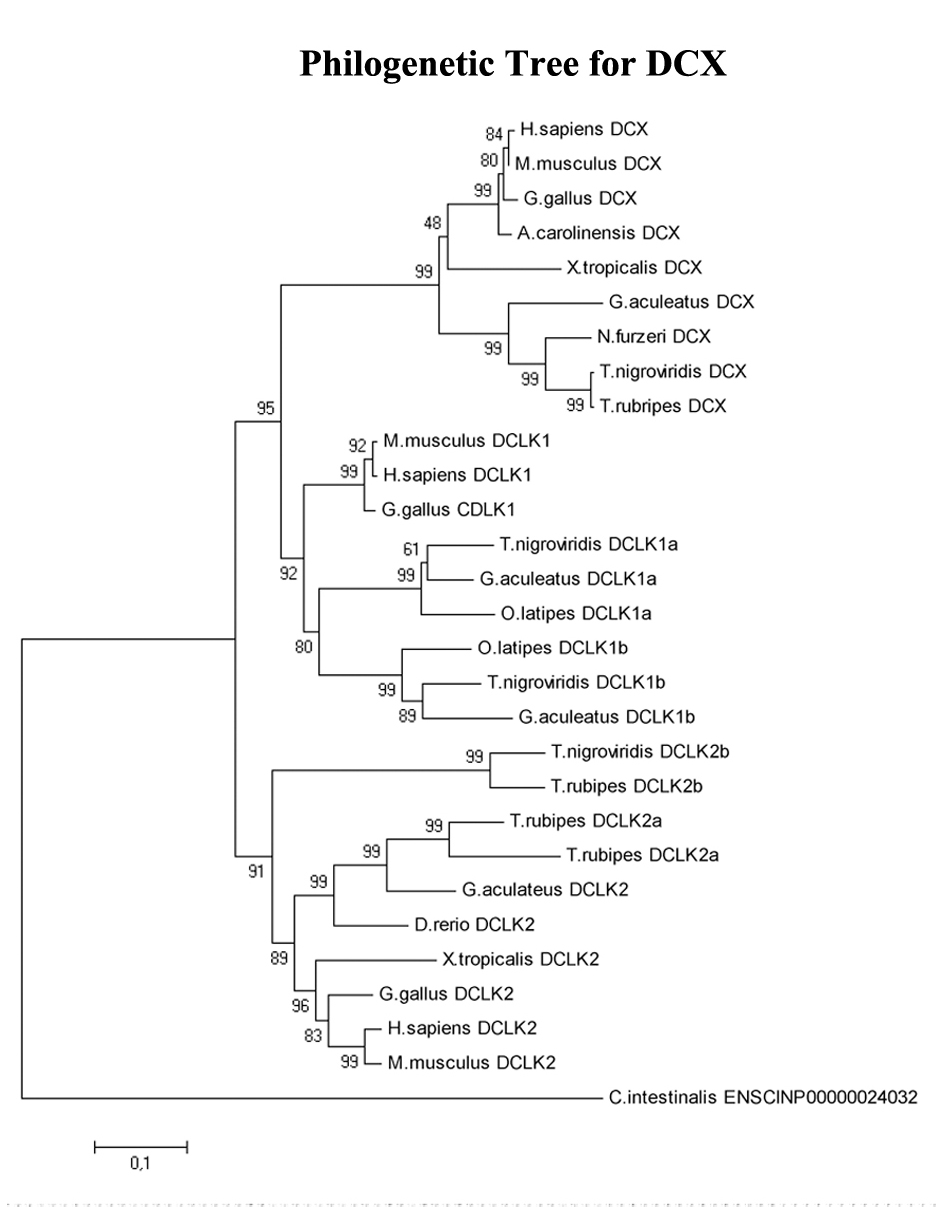

Supplement: Supplementary file 5 [file acel0011-0241-SD5.jpg]

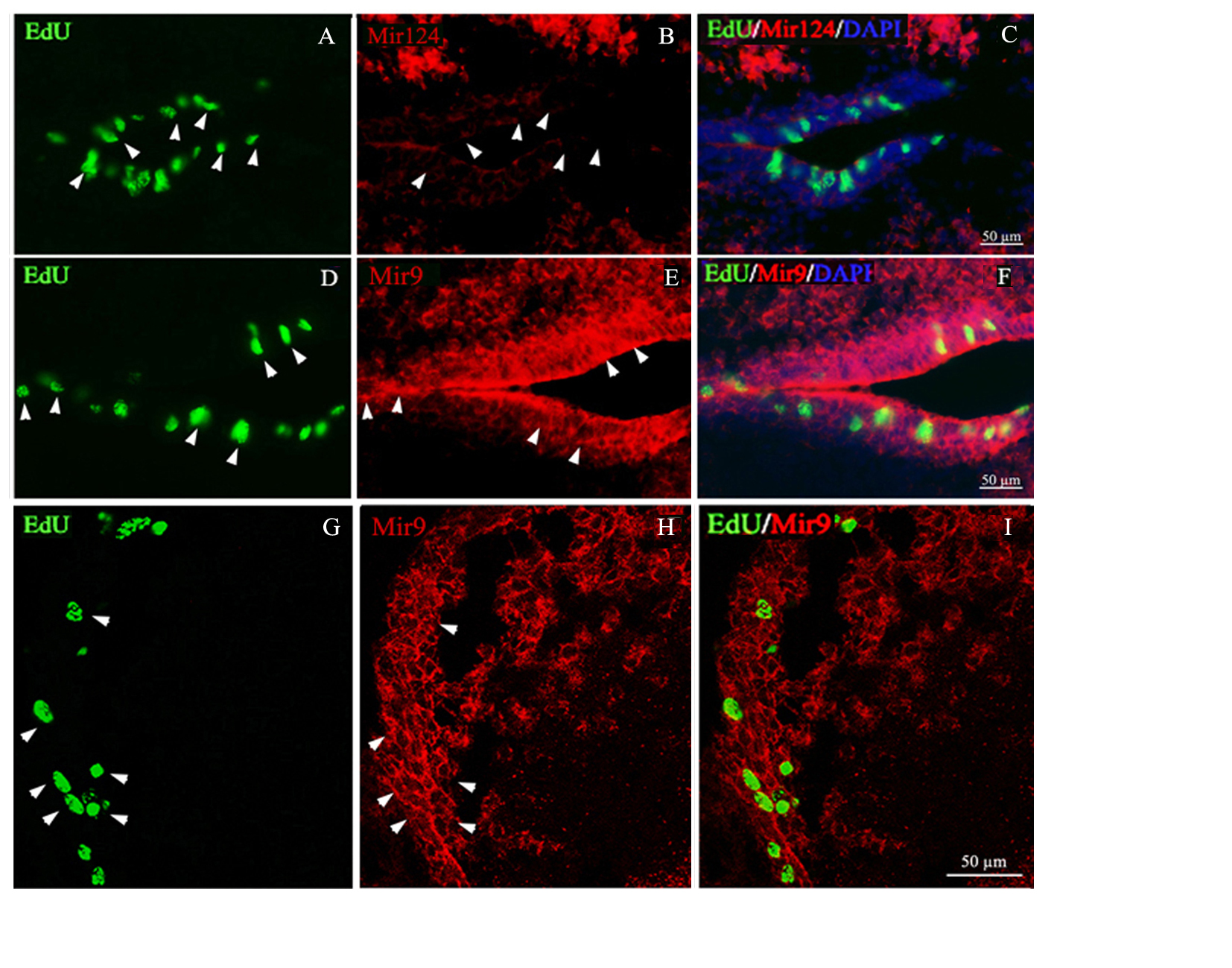

Supplement: Supplementary file 6 [file acel0011-0241-SD6.jpg]

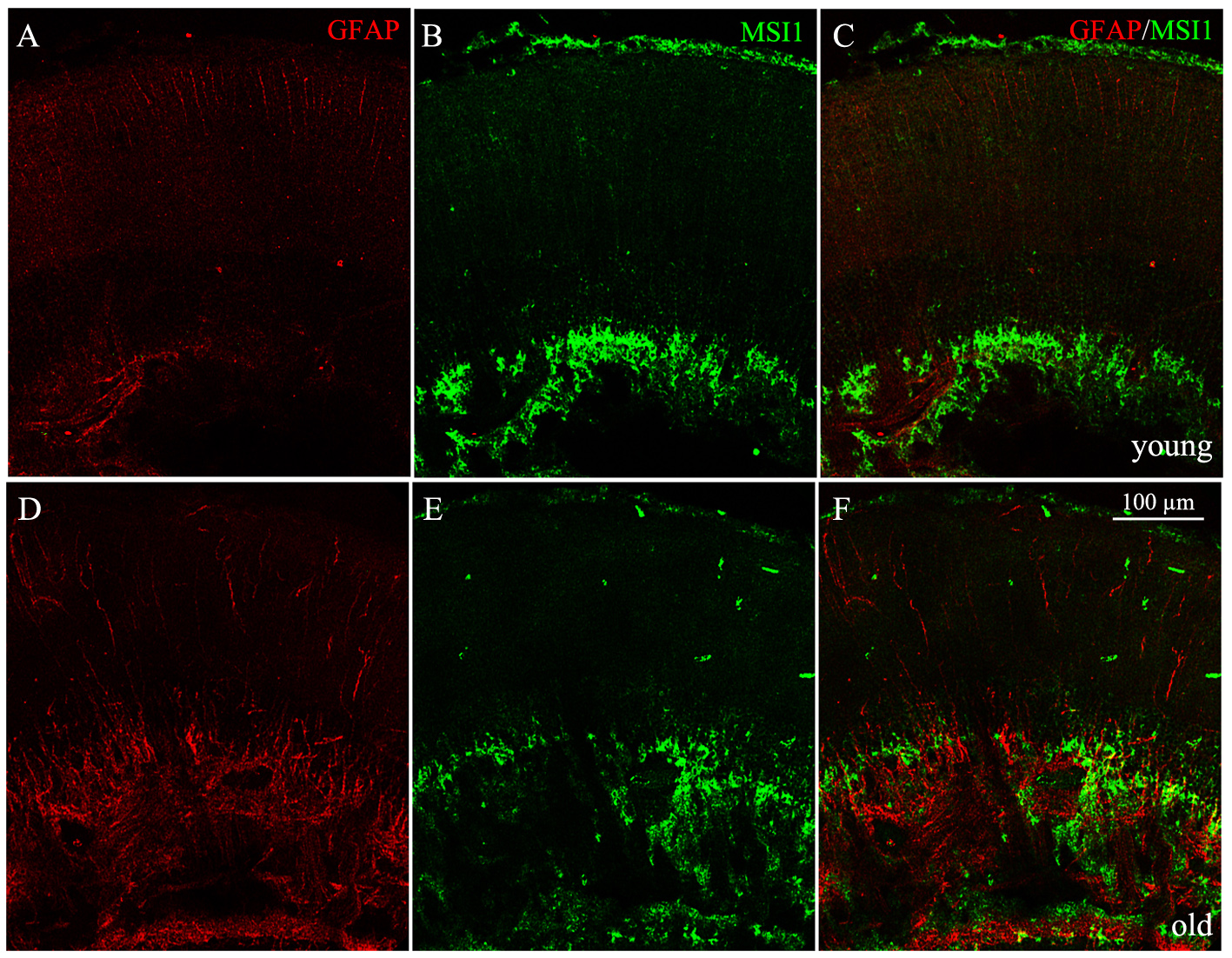

Supplement: Supplementary file 7 [file acel0011-0241-SD7.jpg]

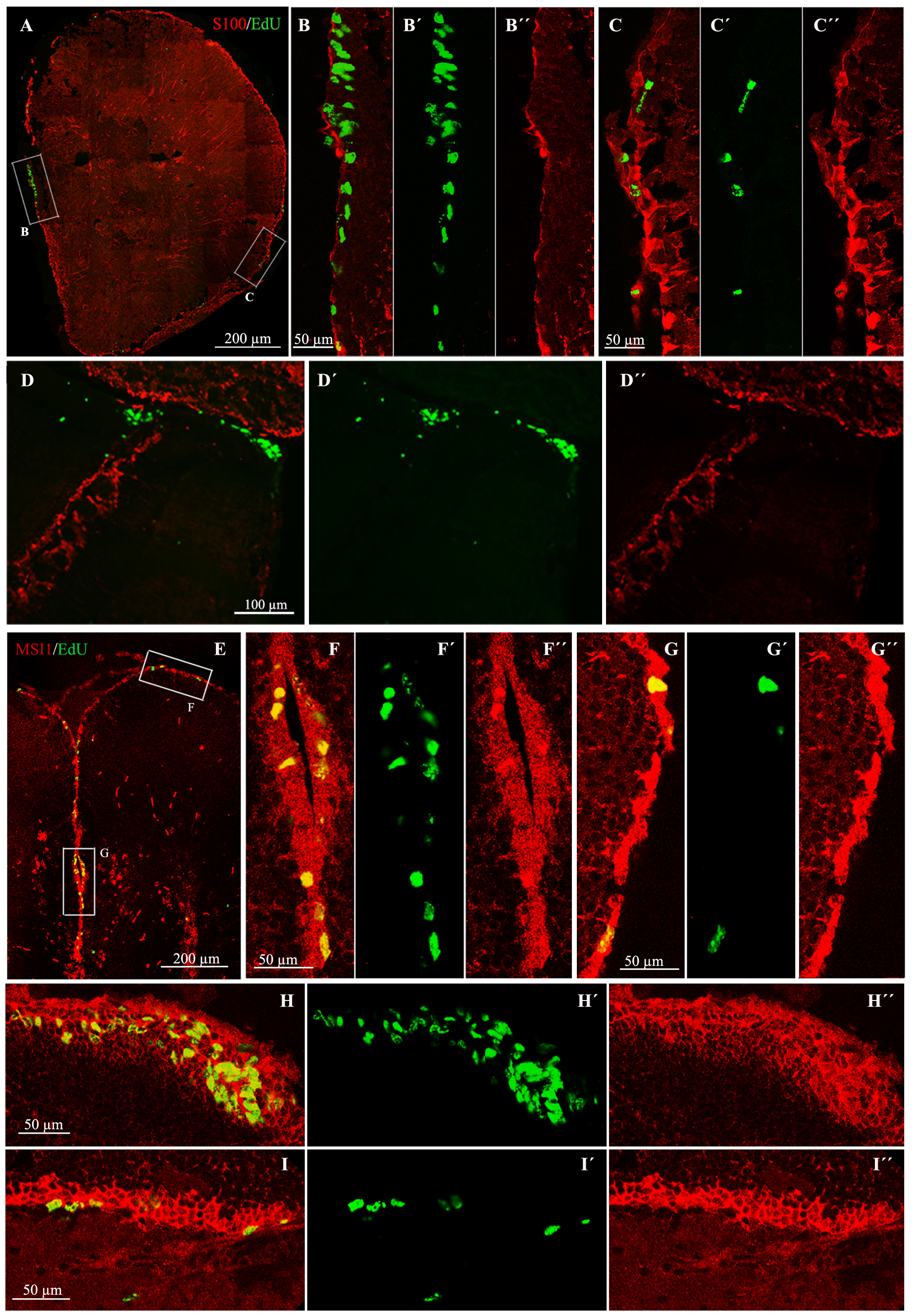

Supplement: Supplementary file 8 [file acel0011-0241-SD8.jpg]
