# Supplementary material for: Adult neurogenesis in the short-lived teleost Nothobranchius furzeri: localization of neurogenic niches, molecular characterization and effects of aging
Source: Aging Cell. 2012 Apr;11(2):241–51. doi: 10.1111/j.1474-9726.2011.00781.x (PMC3437507; doi:10.1111/j.1474-9726.2011.00781.x)

Coronal sections
(complete series ca 50µm -step, rostro- caudal direction)

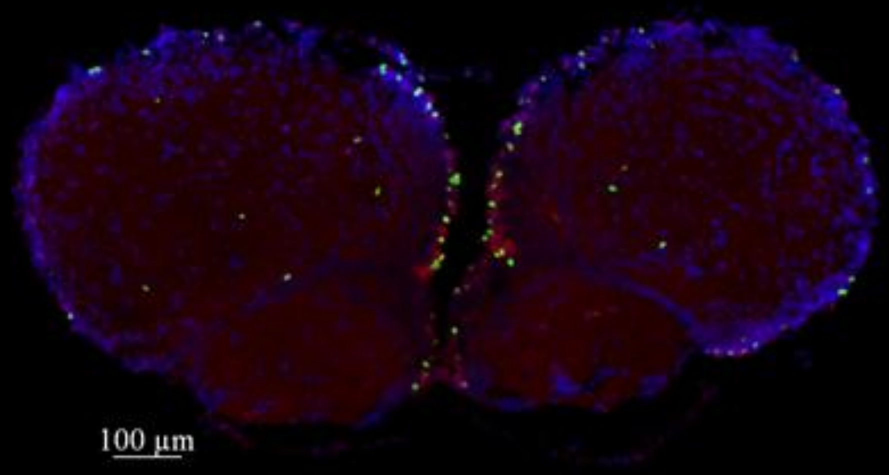

Coronal sections

(complete series ca 50µm -step, rostro- caudal direction)

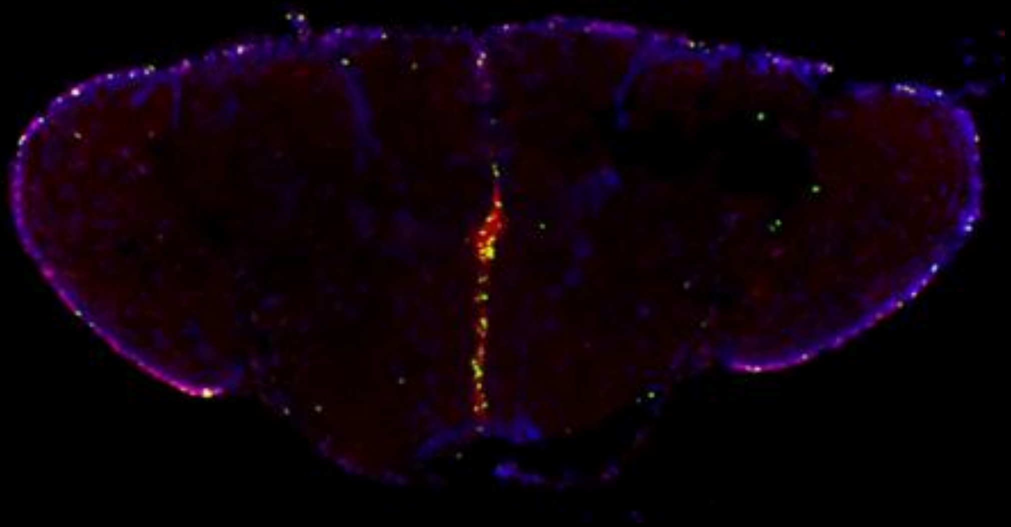

Coronal sections
(complete series ca 50µm -step, rostro- caudal direction)

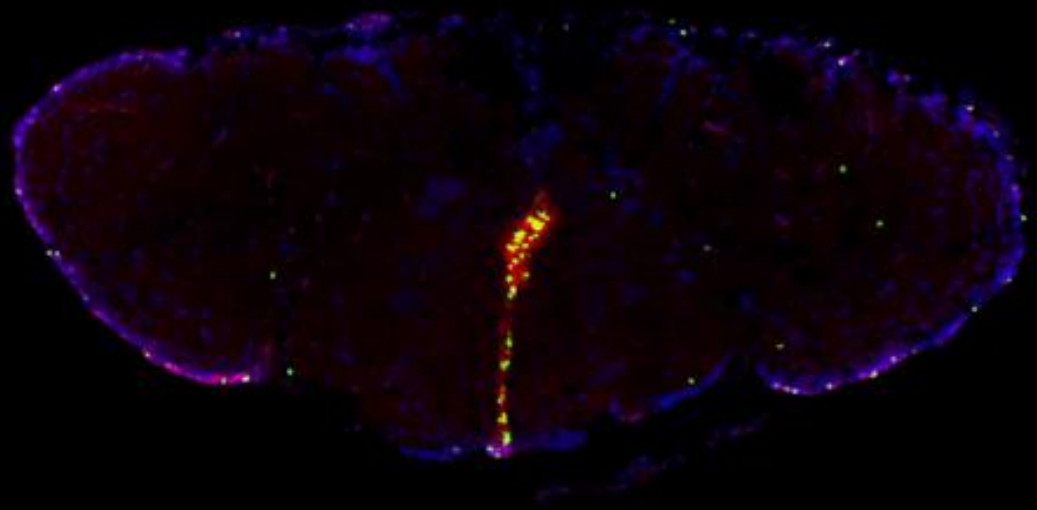

Coronal sections
(complete series ca 50µm -step, rostro- caudal direction)

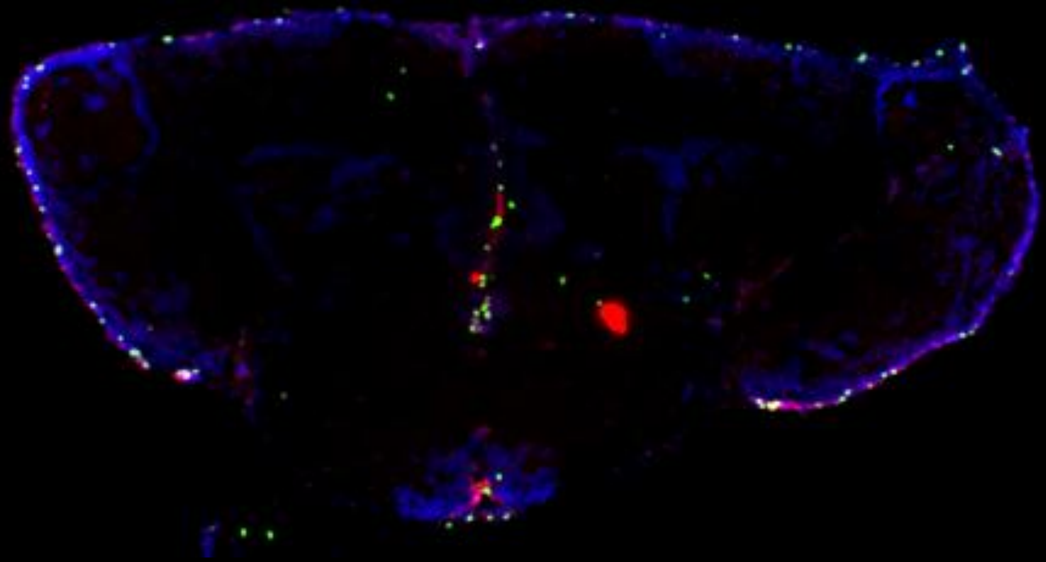

Coronal sections
(complete series ca 50µm -step, rostro- caudal direction)

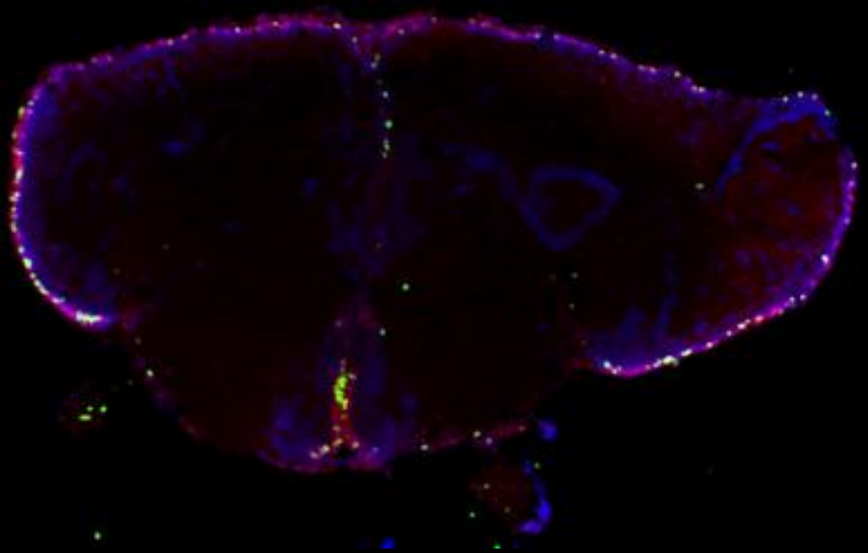

Coronal sections
(complete series ca 50µm -step, rostro- caudal direction)

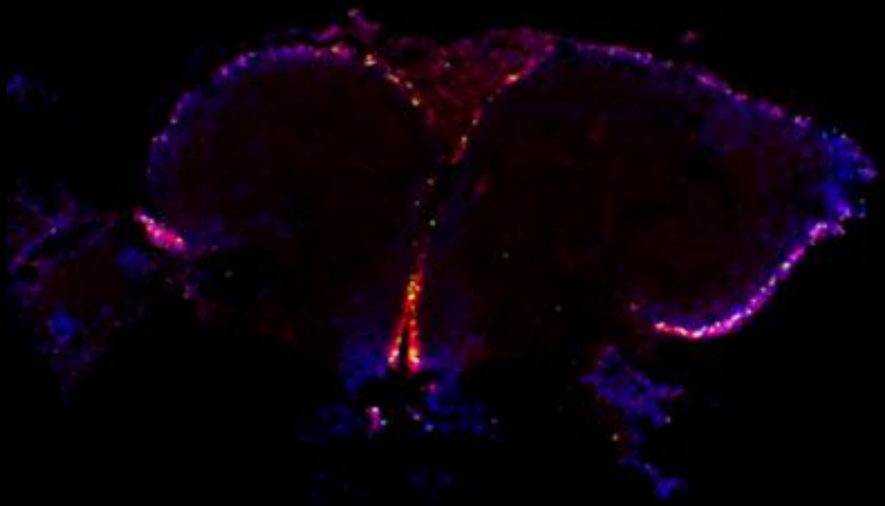

Coronal sections
(complete series ca 50µm -step, rostro- caudal direction)

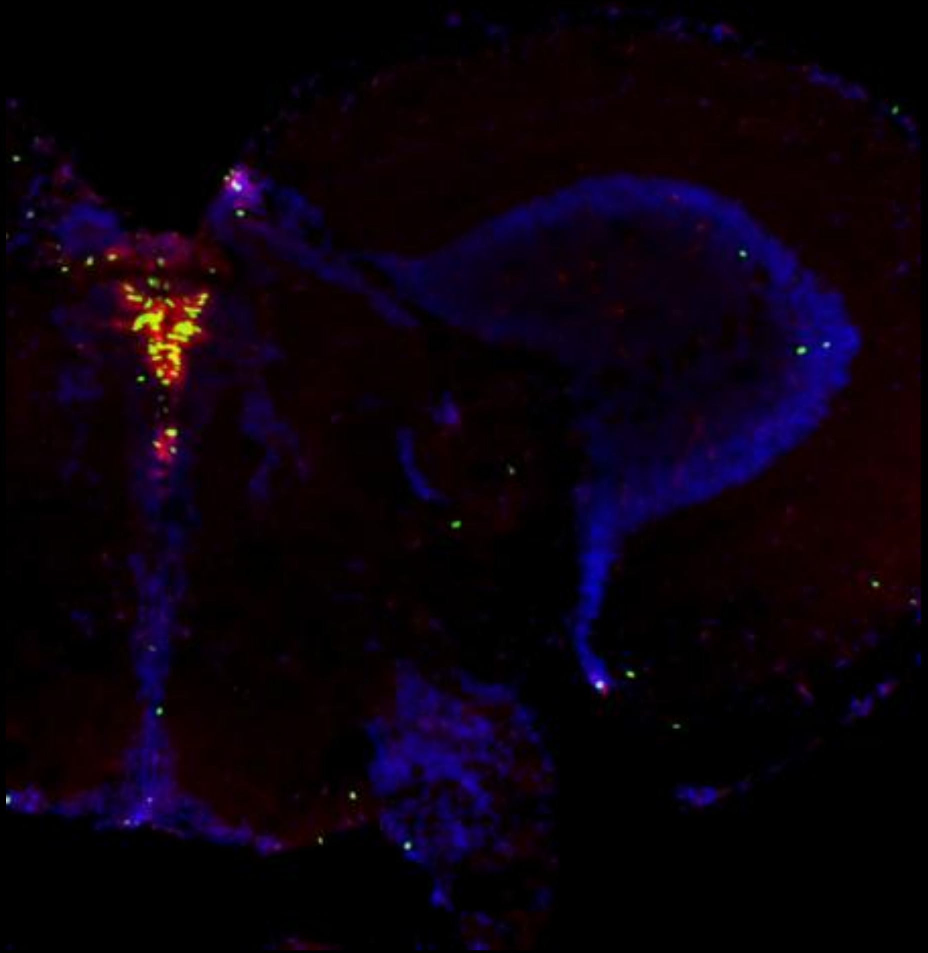

Coronal sections
(complete series ca 50µm -step, rostro- caudal direction)

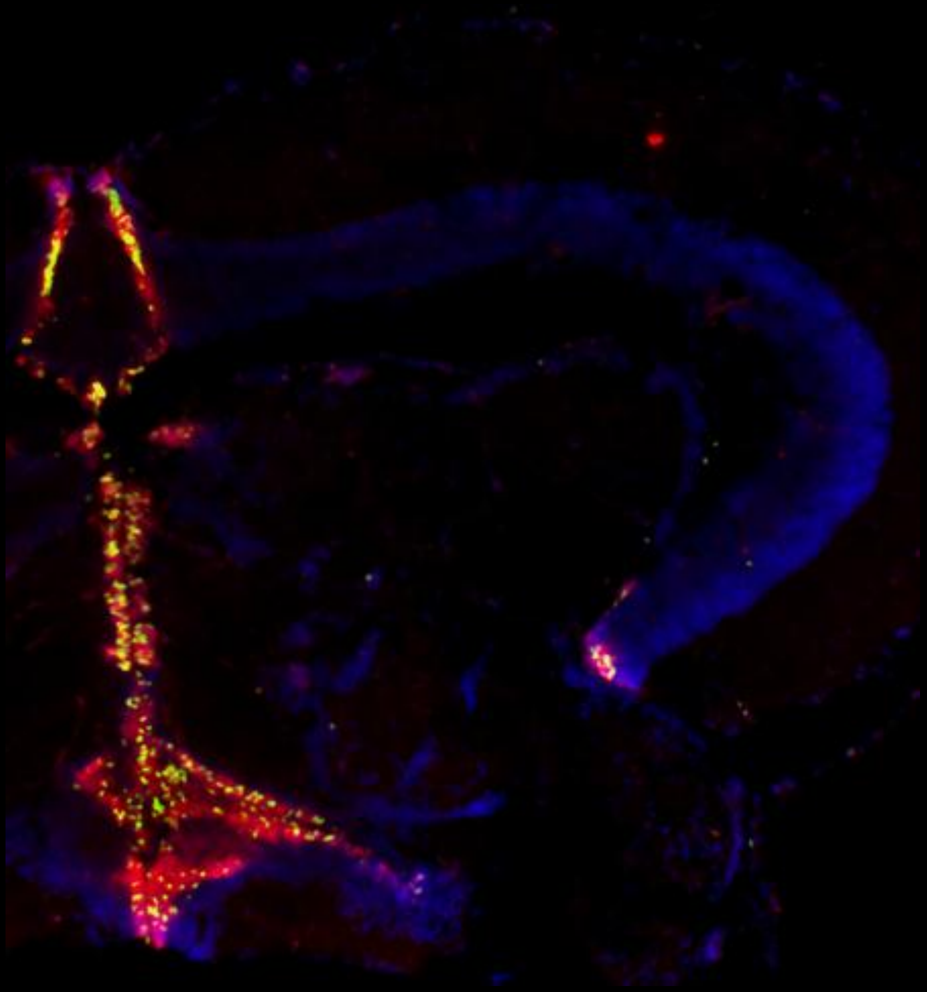

Coronal sections
(complete series ca 50µm -step, rostro- caudal direction)

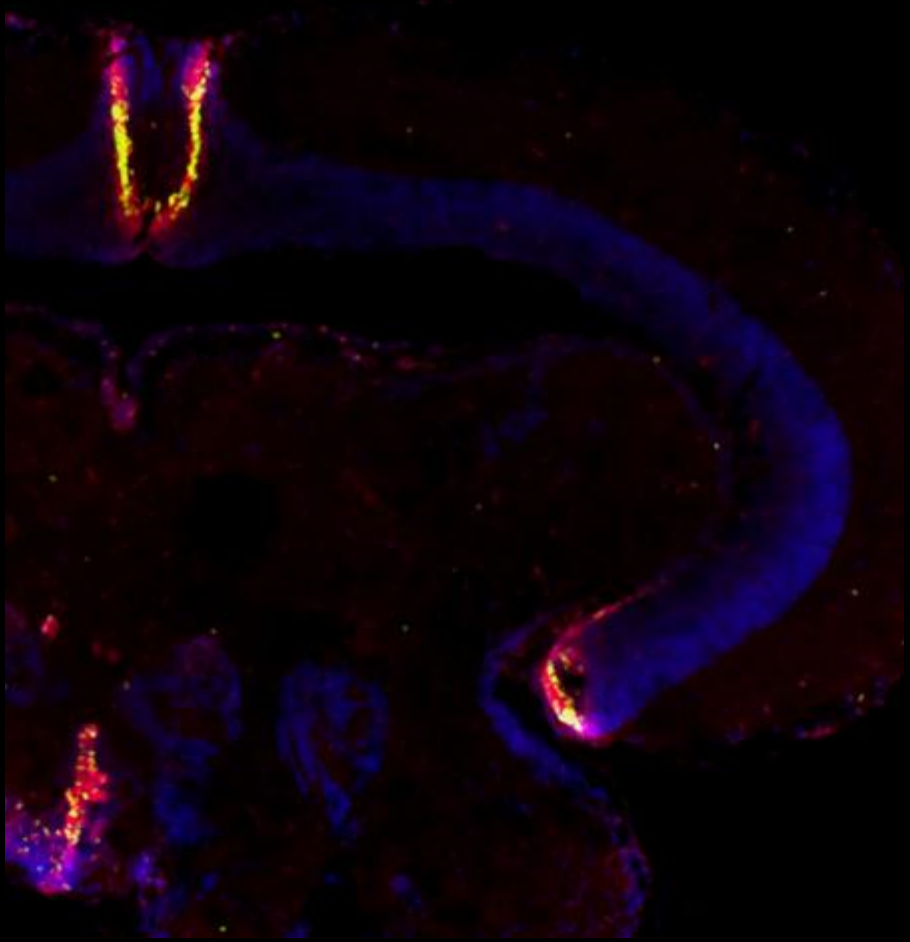

Coronal sections
(complete series ca 50µm -step, rostro- caudal direction)

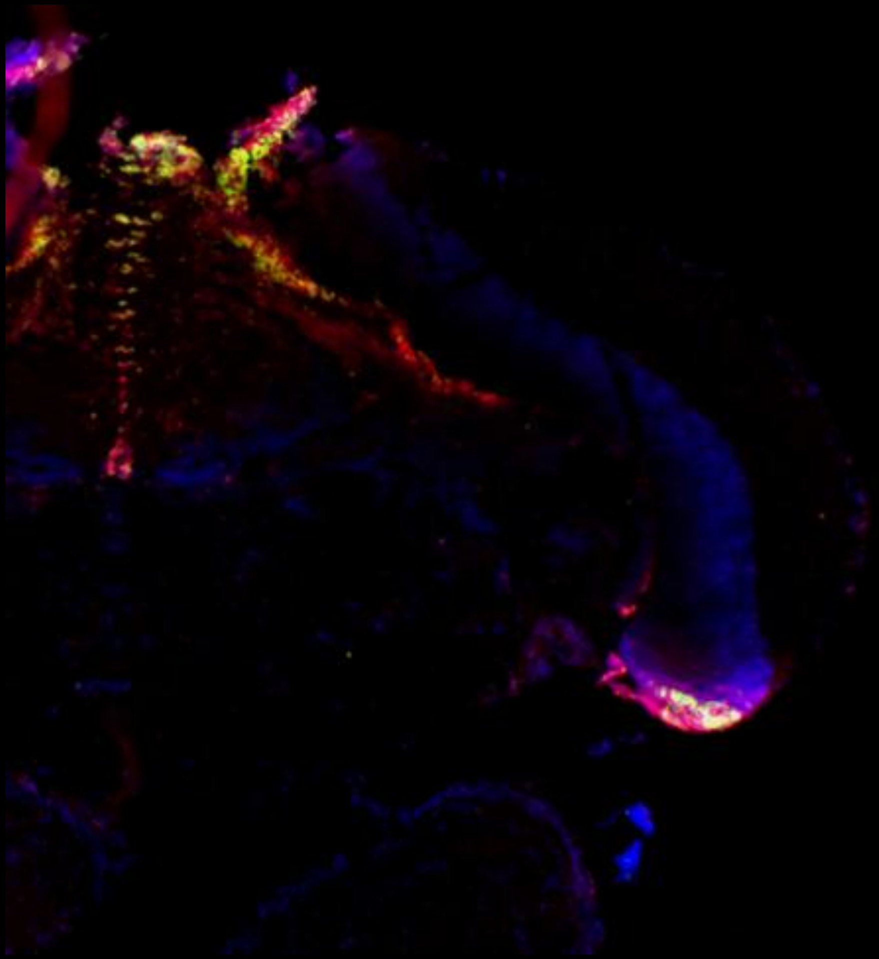

Coronal sections
(complete series ca 50µm -step, rostro- caudal direction)

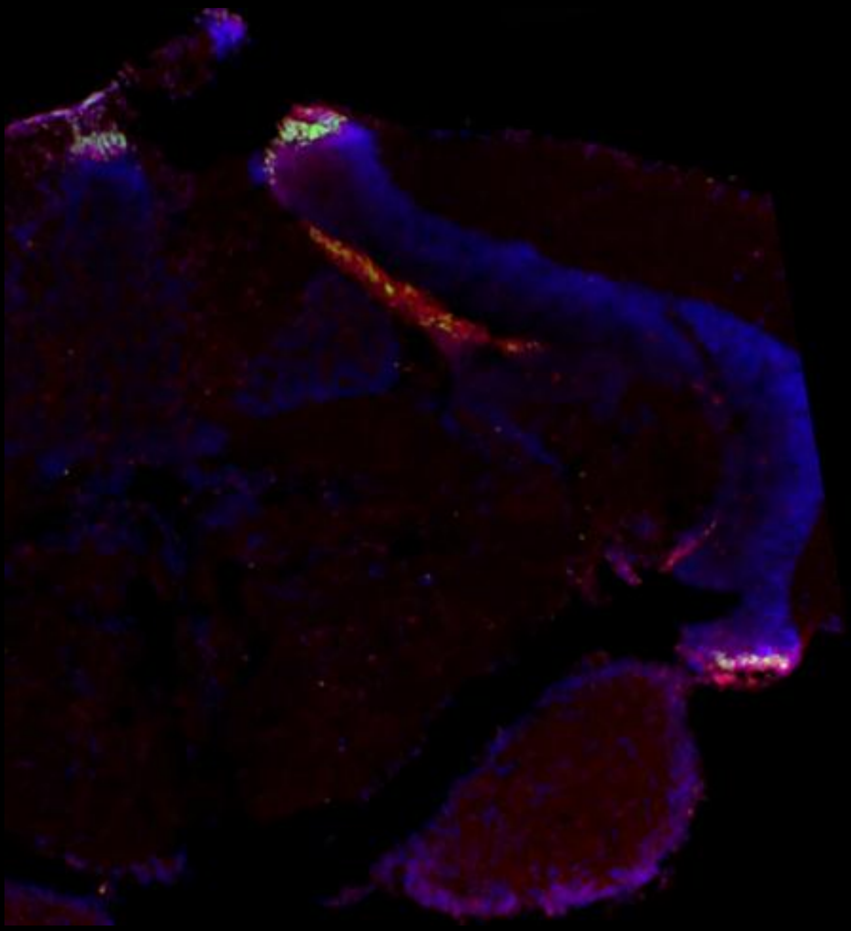

Supplement: Supplementary file 2 [file acel0011-0241-SD2.pdf]
