# Supplementary material for: Adult neurogenesis in the short-lived teleost Nothobranchius furzeri: localization of neurogenic niches, molecular characterization and effects of aging
Source: Aging Cell. 2012 Apr;11(2):241–51. doi: 10.1111/j.1474-9726.2011.00781.x (PMC3437507; doi:10.1111/j.1474-9726.2011.00781.x)
Supplement: Supplementary file 9 [file acel0011-0241-SD9.doc]

**Supplementary Materials and Methods**

**Fish strains and maintenance**

In this paper two strains of *Nothobranchius furzeri* were studied: the longer-lived strain MZM04/10, with medium (27weeks) and maximum (42weeks) lifespan, and the shorter-lived strain GRZ with medium (11weeks) and maximum (15weeks) lifespan (Terzibasi *et al*., 2008 and 2009).

Animal maintenance was performed following German rule for animal experimentation as described (Terzibasi *et al.,* 2009). Fry were fed with newly hatched *Artemia nauplii* for the first 2 weeks and then weaned with finely chopped *Chironomus* larvae. Starting at the fourth week of life, fish were moved to 40 l tanks at a maximum density of 15-20 fish per tank. The temperature was maintained by heating the room at constant 26°C under a light regime of 12:12 h light:dark. Tank water was filtered using air-driven foam filters and twice a week the bottom of the tanks was siphoned and 50% of the water was exchanged.

All experimental subjects were used only after the natural developmental period that is not before 4-5weeks of age. Both strains reach sexual maturity at 4 weeks of age and the large differences in life span are not reflected in large differences for maturation. Details of the aging phenotypes for these strains can be found in Terzibasi *et al*. (2008).

The protocols of animal maintenance and experiments were approved by the local authority in the State of Thuringia (Veterinaer- und Lebensmittelueberwachungsamt).

**Tissue preparation and experimental procedures**

**Edu injectiojn**

Fasted fish were removed from the tank between 8 and 10 A.M., placed on a wet paper bed for the time of the injection and injected intraperitoneally with 10µl of EdU 10mM (PM 252,23) in 0.9% NaCl aqueous solution (providing a fixed dose of 25µg pro fish with 2 to 3gr of body weight > ca 8µgEdu/gr b.w.). The solution was shortly prepared before use. Then animals were replaced in the tank for the time necessary to metabolize EdU. For the injection, we used classical “Hamilton microliter syringes” with the finest (gauge 23) needles.

Whole brains from injected animals were collected at different ages (5, 11, 25 weeks) and time-steps (4h, 1w, 5w) after EdU injection, fixed with PFA 4% o/n, cryoprotected with a 20% and then 30% sucrose solution for at least 3h each. Finally the tissues were embedded at -20°C in Neg50 crio-embedding medium (Thermo scientific); complete series of 16µm thick sections were cut with a Leica cryostat and collected on Superfrost plus slides® (Thermo scientific).

**Immunohistochemistry (IHC)**

Slides were first pr- treated as follows: sections were dried for 2h at 37°C to optimize their fixation on the slides, then placed in a bath of Aceton 100% for 10 minutes at 4°C, air dried for few minutes and washed shortly once in water and twice in PBS at room temperature. As unmasking procedure, slides were placed in a bath of citrate buffer (0.1 M, pH 4.6) and heated in the microwave for 10 minutes at intermediate power, then left to cool at RT and washed again twice with PBS

All sections were blocked with 0.3% Triton X-100, 25% normal goat serum and 5% BSA in PBS, then incubated in the primary antibodies opportunely diluted in a solution made of 10% goat serum, 1% BSA and 0.3% Triton X-100 in PBS), overnight at 4°C.

*Primary antibodies*

List of antibodies and dilutions is provided in Table S1.

:

*Secondary antibodies*

The following secondary antibodies were used: Alexa fluor® 546 (goat anti-rabbit), Alexa fluor® 568 (goat anti-mouse), Alexa fluor® 488 (goat anti-rabbit) and Alexa fluor® 488 (goat anti-mouse), all from Invitrogen (Molecular Probes).

When necessary, EdU detection followed immunolabelling.

**Edu labelling**

We used the Click‑iT® EdU Assay from Invitrogen, a well-known novel alternative to the classical BrdU assay. EdU (5-ethynyl-2´-deoxyuridine) is a nucleoside analog of thymidine and is incorporated into DNA during DNA synthesis, so allowing to label complete populations of mitotically active cells.

Sections were first washed twice shortly in the washing solution used for the entire procedure (3% BSA in PBS), then permeabilized with 20 minutes incubation in the permeabilization buffer (0.5%Triton X-100 in PBS).

Sections were finally labeled for EdU staining without further washings, following the protocol steps explained in the product datasheet of Invitrogen: permeabilized slides were incubated 30 minutes in a fresh prepared EdU staining solution ( “Click-iT® reaction cocktail” ), then washed twice in the washing solution, and again twice in PBS. At this point slides were closed with Fluorcare DAPI® (Roth) in order to visualize cell nuclei in the sections and facilitate the localizations of the different brain regions.

.

**In Situ Hibridization (ISH) on sections:**

*Slide preparation*

Brains were prepared accordingly with the procedure used for IHC and 16 µm thick cryostat sections were either processed immediately or stored at -80°C.

The used ISH protocol is described in detail below:

**1^st^ day**

- Defrost/dry 2hr RT
- Wash 2x 3 min RT
- Proteinase K: dilute 1:20.000 – 1:50.000 (stock 20 microg/microl at -20C °) and use in **PBT** 10 min RT
- Washes 2x 5 min glycine 2mg/ml in PBT at RT
- Washes 2x 3 min in PBT at RT
- PFA 4% 15 min RT
- Washes 3x 3 min in PBT at RT
- Prehybridise 30 min at Thyb in hybridisation buffer (**HB**), using 200 microliter HB, coverslip and humid chamber
- Drain well!! Hybridise at Thyb O/N (150 microliter HB plus probe for each 60mm slides, coverslip, humid chamber). Use 0.3 microliter LNA 3’DIG labelled Exiqon probe .
- Temperature hybridization: 42°C

**2^nd^ day**

- 2x SSC at 42°C to detach coverslip (usually 1-2 min)
- Wash: Twice 2X SSC at RT 10 min, once 0.2X SSC at RT 10 min
- Washes 5x 3 min in PBT at RT
- Block 30 min RT in blocking solution (1% w/v blocking reagent Roche cat. No. 1 096 176 , 1% sheep serum / **MABT**)
- Anti-Dig-AP Fab fragments Ab 1:2000 in blocking solution O/N at 4°C

**3^rd^ day**

- Washes 5x 5 min in PBT at RT
- Washes 3x 5 min in **NMNT** at RT
- Detection RT in darkness (or lower T to slow down the reaction) in Fast Red (Roche tablets; 1 in 2ml 0.1 M Tris-Hcle pH 8.2, vortex 2 min and centrifuge 30 seconds to avoid red precipitate) 20 min - 6 hrs dependignon staining intesity
- Washes 3x 5 min in PBT at RT
- Counterstain (e.g. hoechts or DAPI nuclear staining, immunostaining, EdU detection)

**SOLUTIONS**

**PBT** (prepare fresh)

PBS 1X

0.1% Tween-20

**HB** (store at -20°C)

50% Formamide (Fluka)

5X SSC

0.1% Tween-20

To pH6: Citric acid (460 microl of 1M stock in 50 ml)

50 microg/ml Heparin (Sigma H3393)

500 microg/ml tRNA or torula yeast RNA

**MABT** (store MAB 5X at RT)

100mM Maleic acid

150 mM NaCL

At pH 7.5 with NaOH

0.1% Tween-20

**NMNT** (prepare fresh)

100 mM NaCl

100 mM TrisHCl pH 9.5

50 mM MgCl

0.1% Tween-20

2 mM tetramisole (SIGMA): 500 mg/litre

In situ locked nucleic acid (LNA) probes were labelled with DIG at 5’ and 3’ ends (Exiqon, Denmark) and correspond to the dre-miR-124 and dre-miR-9 sequences deposited in MiRBase.

**Preparation for mRNA expression profiling**

For mRNA expression profiling whole brains from male animals was collected from different time-point. To avoid effects of circadian rhythms and feeding, animals were always sacrificed at 10 a.m. in fasted state. For tissue preparation, fish were euthanized with MS-222 and cooled on crushed ice. The whole fish brains were dissected and transferred into 1.5 ml tubes with 500 µl RNAlater (Qiagen, Hilden, Germany). Tubes were kept at 4°C for one night and then stored at -20°C until RNA extraction.

*RNA extraction*

Total RNA was isolated using QIAzol (Qiagen) according to the manufacturer's protocol, with modifications. In brief, tissue was taken out of the RNAlater, dipped on a clean sterile paper towel to remove residuals of RNAlater and subsequently transferred to a 2 ml sure-lock tube (Eppendorf, Hamburg, Germany). 1 ml cooled QIAzol (Qiagen) and one 5 mm stainless steel bead (Qiagen) was added. Homogenization was performed using a TissueLyzer II (Qiagen) at 20 Hz for 2-3x 1 min. After incubation for 5 min at room temperature 200 µl chloroform was added. The tube was shaken vigorously for (at least) 15 s and incubated for 3 min at room temperature. Phase separation was achieved by centrifugation at 12,000x g for 20 min at 4°C. The aqueous phase was transferred into a fresh cup and 10 µg of Glycogen (Invitrogen, Darmstadt, Germany), 0.16x volume NaAc (2 M; pH 4.0) and 1.1x volume isopropanol were added, mixed thoroughly and incubated for 10 min at room temperature. The RNA was precipitated by a centrifugation step with 12,000 x g at 4°C for 20 min. The supernatant was removed and the pellet was washed with 80% Ethanol twice and air dried for 10 min. The RNA was resuspendet in 20 µl DEPC-treated water by pipetting up and down, followed by incubation at 65°C for 5 min. The RNA was quantified with a NanoDrop 1000 (PeqLab, Erlangen, Germany) and stored at -80°C until use.

**Isolation of *N.furzeri* GFAP**

Isolation of GFAP was obtained by rapid amplification of cDNA ends (RACE) according to the manufacturers protocol (Clonetech).
Brain RNA was isolated from 7 weeks-old male of the GRZ strain.
First strand synthesis:
5' RACE  mix                                           3'RACE mix
3 µl RNA (approx 600 ng)                    3 µl RNA (approx 600 ng)
1 µl 5'CDS-Primer A                             1 µl 3'CDS-Primer A
1 µl smart II Oligo                                 1 µl H2O
  These we mixed, vortexed and spun down, were then incubated for 2 min @ 70°C and then

2 µl 5x first strand buffer

1 µl DTT (20 nm)
1 µl dNTPs (10 mM each)
1 µl MMLV Reverse Transcriptase
Were added and incubated 90 min at 42°C. Then 100 µl TE bufferwas added and incubated for 7 min at 72°C
RACE PCR

2,5 µl cDNA in 25 µl of reaction buffer were amplified using Advantage II Ploymerase using with the following program
prog: 5x(94°C 30s, 72°C 3min),  5x(94°C 30sec, 70°C 30 s, 72°C 3min),
25x(94°C 30s, 68°C 30s, 72°C 3 min)

Primer sequences:
3' RACE
GSP2 (GFAP_f3) GAGGGACAACCTGGCTGCAGA
5'RACE
GSP1 (GFAP_r4) GACGTTGAGAAGGTCCTGGTA

Nested RACE
for Nested PCR, the Products from PCR1 were diluted: 5 µl PCR Product to
245 TE Buffer
PCR setup was as discribed on page 23, point 6
Primer:
3' RACE
NGSP2 (GFAP_f4) CATGATGAGGAGCTGCGTGAGTT
5'RACE
NGSP1 (GFAP_r3) CGCCGGTACTCATTGGCCTC

**Quantitative real-time PCR**

Real-time PCR was performed with the RotorGene 6000 and the miScript PCR system (both Qiagen). Steps were processed as recommended by the manufacturer. The miScript Reverse Transcription kit (Qiagen) was used to convert RNA to cDNA using oligo dT and random primers. The miScript RT mix is an optimized blend of enzymes comprising a poly(A) polymerase and a reverse transcriptase and is optimised to analyse precursor miRNA, mature miRNA, other small non-coding RNA, and mRNA in the same sample. We used 500 ng total RNA for cDNA sysnthesis in a 20 µl Volume. After cDNA synthesis samples were diluted to a final volume of 200 µl with ultra pure water (to an approximate cDNA concentration of 40 ng/µl). PCR reactions were performed in 20 µl volume with 2 µl diluted cDNA using the Quantitect SYBR Green PCR kit (Qiagen). A cDNA pool was serially diluted in steps of 2 (from 80 to 2.5 ng per reaction) and used to create standard as well as melting curves and to calculate amplification efficiencies for each primer prior to use for quantification. All reactions were performed in triplicates and negative (water) as well as genomic (without reverse transcriptase) controls were always included. Fold changes describe the difference in expression level between young and old age animals normalised to the stable reference mir-124, which was not significantly changed between age groups based on our sequencing results. Statistical analysis of real-time data was performed with the relative expression software tool REST (Qiagen, Pfaffl et al., 2002). This software tool uses a mathematical model that compares unknown and control samples and significance is tested by a randomisation test (pair wise fixed reallocation randomisation test).

Sequences of the primers used:

Hprt_IntronB_fw CACTCTGACAGGCAAGAATG
Hprt_IntronB_rev ACACTCCGTGGTGTTCTCTT
Tbp_IntronF_fw CGGTTGGAGGGTTTAGTCCT
Tbp_IntronF_rev GCAAGACGATTCTGGGTTTG
GFAP_RT_f2_E4 AACCTGCAGGAGACAGAGGA
GFAP_RT_r2_E6 CCGTGTCCTGATAACCTGCT
PCNA_RT_f4_E1 CAGAGCATGGACTCCTCTCA
PCNA_RT_r4_E2 TCCTCTGCTCTGAGGGTGAT

HPRT ( ) and/or TBP ( ) were used as normalization

**Phylogenetic analysis**

Phylogenetic analysis was performed is MEGA5 (Tamura et al., 2011). All protein sequences were downloaded from ENSEMBL aligned using ClustaW and the alignment manually inspected. Several different algorithms and substitutions models were tested and all retrieved the same topology with NfuDCX nested within all other DCX sequences. Interior branch test was used to calculate the support for the branches.

**Image acquisition, cell countings and statistics**

Immunostainings were documented with a fluorescence microscope (Carl Zeiss HBO 50/AC) associated with the 20x and 40x (dry) objectives and the AxioVision 4.2 program, and two confocal microscopes: a Carl Zeiss LSM510 associated with the 20x, 40x (dry) and the 63x (oil-immersion) objectives and the LSM510 Image Program, and a Leica DMIRE2 with 20x (dry) and 40x, 63x (oil-immersion) objectives and the Leica Confocal program

*EdU^+^ cells quantification in the optic tectum*

To quantify the total number of EdU^+^ cells through the entire thickness of the tectal structure we used an adaptation of the “Optical Fractionator Method” (OFM), the most commonly applied stereological probe to estimate the total number of objects in a three dimensional volume. Here we sampled the full tickness of the tectal structures of 5weeks- and 25weeks- old fish brains (five subject per age-group) by collecting horizontal cryo-sections 16 µm thick along the dorso-ventral axis, and we focused the quantitative analysis on the proliferative niche marked as region VI (Fig 2A); every third section entered into the stastistical analysis and confocal (single optical plane) images of region VI were acquired with a Z-step of 4 µm (mean diameter of the EdU^+^ cells) trhrough the full section. Two consecutive optical plans for each analyzed section were visualized by Photoshop with different colours (red and green) and overlapped, to exclude from the conting all double labelled cells (yellow) and so avoiding EdU^+^ cells overextimation. Then, the extimation of the total number of EdU^+^ cells into the tectal region VI was calculated by using the formula described below.

*Unbiased estimates:* If an ordered series of sections is
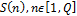
, and it is decided for a sample density of
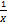
 , where
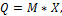
 then a random number
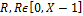
 is extracted and every section
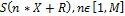
 is analyzed. Then the unbiased estimate of cell numbers
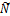
can be calculated by the formula


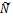
=
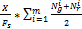
 , where:


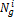
 = number of green profiles in section
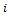


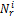
 =number of red profiles in section
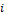


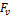
=fraction of volume sampled within one section.

**Quantification of GFAP and S100B immunohistochemistry**

Fluorescence intensity was quantified following a semiquantitative method: first,double-labelled images were acquired using a Zeiss LSM confocal microscope at an excitation wavelength of 488 nm (S100B) and 547 nm (GFAP), with fixed imaging parameters (magnification, pinhole, photomultiplier,laser intensity, etc.). After acquisition, autofluorescent erithrocytes were manually erased using Adobe Photoshop.

Erythrocytes could be unambiguously identified based on two characteristics: (i) their typical nucleated morphology and (ii) their location within blood vessels.

Curated images were then analysed using ImageJ (http://rsbweb.nih.gov/ij/) to quantify percentage

of area over threshold. During thresholding, all pixels below a defined greyscale value are transformed into 0 and all pixels above it are transformed into 1. The threshold level is defined once by the experimenter so to exclude all background labelling and is then kept constant for all images to be compared. The percentage of pixels with value 1 in the image is the metric used for the statistic. Three sections and three images in the central optic tectum were quantified. Values relative to GFAP labeling were then divided by the values relative to S100B labelling on the same slide.
