# Supplementary material for: Adult neurogenesis in the short-lived teleost Nothobranchius furzeri: localization of neurogenic niches, molecular characterization and effects of aging
Source: Aging Cell. 2012 Apr;11(2):241–51. doi: 10.1111/j.1474-9726.2011.00781.x (PMC3437507; doi:10.1111/j.1474-9726.2011.00781.x)
Supplement: Supplementary file 10 [file acel0011-0241-SD10.doc]

Alignment of NfuDCX with mouse DCX (gi|123226162|emb|CAM25091.1|)

Score = 1430 (508.4 bits), Expect = 2.2e-146, P = 2.2e-146

Identities = 284/365 (77%), Positives = 315/365 (86%), Frame = +2

Query: 1 MELDFGHFDERDKASRNMRGSRMNGLPSPTHSAHCSFYRTRTLQALSNEKKAKKVRFYRN 60

MELDFGHFDERDK SR RG RMNGLPSPTHSAHCSFYRTRTLQAL+NEKKAKKVRFYRN

Sbjct: 119 MELDFGHFDERDKTSRTPRGGRMNGLPSPTHSAHCSFYRTRTLQALTNEKKAKKVRFYRN 298

Query: 61 GDRYFKGIVYAVSSDRFRSFDALLADLTRSLSDNINLPQGVRYIYTIDGSRKIGSMDELE 120

GDRYFKGIVYAV++DRFR+FD+LLADLTRSL+D+INLPQGVR+I+TIDGSRKI ++DELE

Sbjct: 299 GDRYFKGIVYAVANDRFRTFDSLLADLTRSLADHINLPQGVRFIFTIDGSRKISTLDELE 478

Query: 121 EGESYVCSSDNFFKKVEYTKNVNPNWSVNVKTSANMKAPQSLASSNSAQARENKDFVRPK 180

EGESYVC+S+NF+KKV+YTKNVNPNWSVNVK SA+ K QSLA+ + RE KDFVRPK

Sbjct: 479 EGESYVCASENFYKKVDYTKNVNPNWSVNVKASASQKNMQSLAAKAAGDPREGKDFVRPK 658

Query: 181 LVTIIRSGVKPRKAVRVLLNKKTAHSFEQVLTDITEAIKLETGVVKKLYTLDGKQVTCLH 240

LVT++RSGVKPRKAVRVLLNKKTAHSFEQVLTDITEAIKLE+GVVKK+YTLDGKQVTCL

Sbjct: 659 LVTVMRSGVKPRKAVRVLLNKKTAHSFEQVLTDITEAIKLESGVVKKIYTLDGKQVTCLQ 838

Query: 241 DFFGDDDVFIACGPEKFRYAQDDFSLDENECRVMKGNPSAAAGPKASPTPQKTSAKSPGP 300

DFFGDDDVFIACGPEKFRYAQDDFSLDENECR+MK + KA Q S KSPGP

Sbjct: 839 DFFGDDDVFIACGPEKFRYAQDDFSLDENECRLMKTS-------KA----QHGSYKSPGP 985

Query: 301 MRRSKSPADSGNDQDANGTSSSQLSTPKSKQSPISTPTSPGSLRKHKDLYLPLSLDDSDS 360

++ SKSP +S N SSSQ+STP SK SP S+PTSPG K KDL LPLSL+D DS

Sbjct: 986 IKCSKSP-ESTNGT----ASSSQISTPISKHSPTSSPTSPGPNNKQKDLDLPLSLEDEDS 1150

Query: 361 LGDSM 365

LG+SM

Sbjct: 1151 LGESM 1165
