# Supplementary material for: Adult neurogenesis in the short-lived teleost Nothobranchius furzeri: localization of neurogenic niches, molecular characterization and effects of aging
Source: Aging Cell. 2012 Apr;11(2):241–51. doi: 10.1111/j.1474-9726.2011.00781.x (PMC3437507; doi:10.1111/j.1474-9726.2011.00781.x)
Supplement: Supplementary file 11 [file acel0011-0241-SD11.doc]

Alignment of NfuGFAP with mouse GFAP (gb|AAK56091.1|AF332062_1)

Score = 465 bits (1197), Expect = 2e-164

Identities = 237/345 (69%), Positives = 289/345 (84%), Gaps = 5/345 (1%)

Frame = +1

Query 205 RLDFSADSLLKAQYKETRTNEKMEMMGLNDRFASYIEKVRLLEQQNKVLVAELNQLKGKE 384

R+DFS L A +KETR +E+ EMM LNDRFASYIEKVR LEQQNK L AELNQL+ KE

Sbjct 16 RVDFSLAGALNAGFKETRASERAEMMELNDRFASYIEKVRFLEQQNKALAAELNQLRAKE 75

Query 385 PSRLGDIYQMELRDLRRQVDDLTNGKARLEIERDNLSADLGTLKQRLQDEMGLRQEAENS 564

P++L D+YQ ELR+LR ++D LT ARLE+ERDNL+ DLGTL+Q+LQDE LR EAEN+

Sbjct 76 PTKLADVYQAELRELRLRLDQLTANSARLEVERDNLAQDLGTLRQKLQDETNLRLEAENN 135

Query 565 LNAFRQDVDEAALSRVQLERKIEALQDEINFLKKIHEEELRELQEQIMAQQVHVDVDVSK 744

L A+RQ+ DEA L+RV LERK+E+L++EI FL+KI+EEE+REL+EQ+ QQVHV++DV+K

Sbjct 136 LAAYRQEADEATLARVDLERKVESLEEEIQFLRKIYEEEVRELREQLAQQQVHVEMDVAK 195

Query 745 PDLTAALRDIRVQYENMATSNLQETEDWYRSKFADLTDAANRNAEALRQAKQEANDYRRQ 924

PDLTAALR+IR QYE +ATSN+QETE+WYRSKFADLTDAA+RNAE LRQAK EANDYRRQ

Sbjct 196 PDLTAALREIRTQYEAVATSNMQETEEWYRSKFADLTDAASRNAELLRQAKHEANDYRRQ 255

Query 925 VQVLTCDLDALRGTNESLERQLRELEDRCAMETAGYQDTASRLEEEIQTLKEEMARHLQE 1104

+Q LTCDL++LRGTNESLERQ+RE E+R A E+A YQ+ +RLEEE Q+LKEEMARHLQE

Sbjct 256 LQALTCDLESLRGTNESLERQMREQEERHARESASYQEALARLEEEGQSLKEEMARHLQE 315

Query 1105 YQDLLNVKLALGIEIHAYRKLLDGKVVRIYA-----TNLRVQNSS 1224

YQDLLNVKLAL IEI YRKLL+G+ RI +NL+++ +S

Sbjct 316 YQDLLNVKLALDIEIATYRKLLEGEENRITIPVQTFSNLQIRETS 360
