# Supplementary material for: Adult neurogenesis in the short-lived teleost Nothobranchius furzeri: localization of neurogenic niches, molecular characterization and effects of aging
Source: Aging Cell. 2012 Apr;11(2):241–51. doi: 10.1111/j.1474-9726.2011.00781.x (PMC3437507; doi:10.1111/j.1474-9726.2011.00781.x)
Supplement: Supplementary file 12 [file acel0011-0241-SD12.doc]

| Gene | Species | Accession number |
| --- | --- | --- |
| DCX | *H.sapiens* | ENSP00000348553 |
| DCX | *M.musculus* | ENSMUSP00000108477 |
| DCX | *G.gallus* | ENSGALP00000031683 |
| DCX | *A.carolinensis* | ENSACAP00000013078 |
| DCX | *X.tropicalis* | ENSXETP00000027143 |
| DCX | *G.aculeatus* | ENSGACP00000027378 |
| DCX | *T.nigroviridis* | ENSTNIP00000012945 |
| DCX | *T.rubipes* | ENSTRUP00000042951 |
| DCLK-1 | *M.musculus* | ENSMUSP00000050034 |
| DCLK-1 | *H.sapiens* | ENSP00000255448 |
| DCLK-1 | *G.gallus* | ENSGALP00000027501 |
| DCLK-1 | *T.nigroviridis* | ENSTNIP00000012816 |
| DCLK-1 | *O.latipes* | ENSORLP00000004902 |
| DCLK-1 | *G.aculeatus* | ENSGACP00000027182 |
| DCLK-1 | *O.latipes* | ENSORLP00000004577 |
| DCLK-1 | *T.nigroviridis* | ENSTNIP00000019361 |
| DCLK-1 | *G.aculeatus* | ENSGACP00000015328 |
| DCLK-2 | *T.nigroviridis* | ENSTNIP00000014188 |
| DCLK-2 | *T.rubipes* | ENSTRUP00000044784 |
| DCLK-2 | *T.nigroviridis* | ENSTNIP00000020883 3 |
| DCLK-2 | *T.rubipes* | ENSTRUP00000038153 |
| DCLK-2 | *G.aculeatus* | ENSGACP00000022702 |
| DCLK-2 | *D.rerio* | ENSDARP00000010634 |
| DCLK-2 | *X.tropicalis* | ENSXETP00000040906 |
| DCLK-2 | *G.gallus* | ENSGALP00000027502 |
| DCLK-2 | *H.sapiens* | ENSP00000303887 |
| DCLK-2 | *M.musculus* | ENSMUSP00000096581 |
| Outgroup | *C.intestinalis* | ENSCINP00000024032 |

Supplementary table I: list of accession numbers for the sequences used to create the phylogenetic tree reported in Fig. 6
