# Supplementary material for: Adult neurogenesis in the short-lived teleost Nothobranchius furzeri: localization of neurogenic niches, molecular characterization and effects of aging
Source: Aging Cell. 2012 Apr;11(2):241–51. doi: 10.1111/j.1474-9726.2011.00781.x (PMC3437507; doi:10.1111/j.1474-9726.2011.00781.x)
Supplement: Supplementary file 13 [file acel0011-0241-SD13.doc]

| Antigen Specificity | Company | Origin | Dilution | Working conditions | Cell types labelled |
| --- | --- | --- | --- | --- | --- |
| PCNA | Dako  Santa Cruz | Mouse Monoclonal  Rabbit policlonal | 1:1000  1:50 | 24h, 4°C  24h, 4°C | Mitotically active cells |
| HuC/D | Invitrogen | Mouse Monoclonal | 1:50 | 24h, 4°C | Differentiated neurons |
| S100B | Dako | Rabbit Policlonal | 1:400 | 24h, 4°C | Radial Glia |
| Musashi-1 (Msh1) | Cell  Signalling | Rabbit Monoclonal | 1:100 | 48h, 4°C | Radial glia and neuroblasts |
| Glial Fibrillary Acid Protein (GFAP) | Sigma | Mouse Monoclonal | 1:400 | 24h, 4°C | Radial Glia |
| Doublecortin (Dcx) | AbCam | Rabbit policlonal | 1:200 | 48h, 4°C | New born neurons |
